# Individual differences in sharing false political information on social media: Deliberate and accidental sharing, motivations and positive schizotypy

**DOI:** 10.1371/journal.pone.0304855

**Published:** 2024-06-26

**Authors:** Tom Buchanan, Rotem Perach, Deborah Husbands, Amber F. Tout, Ekaterina Kostyuk, James Kempley, Laura Joyner

**Affiliations:** 1 School of Social Sciences, University of Westminster, London, United Kingdom; 2 School of Psychology, University of Sussex, Sussex, United Kingdom; University of Belgrade Faculty of Philosophy: Univerzitet u Beogradu Filozofski Fakultet, SERBIA

## Abstract

False political information–misinformation or disinformation—is widely spread on social media. Individual social media users play a large part in this. However, only a minority actively share false material. It is important to establish what sets these individuals apart from those who do not, and why they do it. Motivations for sharing may vary and are likely to differ between people who share false material unknowingly and on purpose. In this paper we consider the extent to which individual differences in personality and other variables, and motivations for sharing, are associated with the likelihood of people sharing false political information both accidentally and deliberately. In a series of four studies (*N*s = 614, 563, 627, 113) we examined predictors of sharing false political information using different methodological approaches. Across the four studies, a key finding was that positive schizotypy is associated with measures of sharing false information both accidentally and deliberately. Motivations for sharing political information online were also relevant, with sharing for reasons of ’raising awareness’ appearing particularly important. Implications for research and practice are discussed.

## Introduction

False political information is shared widely online [1], potentially contributing to profoundly harmful outcomes including political and social unrest and violence [e.g. 2], reduction of trust in genuine political material [e.g. 3], and increased belief in conspiracy theories including harmful false ideas about health, climate change and politics [e.g. 4]. Human action is a key element in how false information spreads [5]. Individuals may directly share material to their own networks of contacts, or engage with it in different ways that prompt algorithmic propagation through the mechanisms of social network platforms [6]. People may share false information deliberately [7], with an intent to cause harm (disinformation), or in the mistaken belief that it is true (misinformation).

Only a minority of social media users actively share false information. It is important to investigate what sets those who share false information apart from those who do not, for two main reasons.

The first is to increase our understanding of why people do this. Research to date suggests the reasons people share false information include personality and other individual differences [1]. For example, some work has shown that false information was largely spread by individuals with conservative political views who were also low on the personality trait of conscientiousness [8].

The second is to understand whether and how individual differences may bear on attempts to do something about the problem. Considerable effort has been invested in devising interventions to minimize the spread or influence of false information. These include focusing attention on accuracy [9], raising digital media literacy [10], and ‘inoculation’ procedures [e.g. 11]. While all these approaches can work, there is evidence that interventions may not be equally useful for all individuals. For example, while attention-focusing interventions are generally seen as effective, there have been suggestions that they may be less so for some individuals with right-wing political views [12, 13]. Beyond characteristics such as political orientation, the motives of individuals spreading false information are likely to be important. Estimates vary, but somewhere in the region of 10–40% of social media users may report having shared such material [14–16]. In many cases this is done unwittingly: some people will share material they come across in the belief that it is true. However, some individuals–perhaps half of those sharing false material–do it in the full knowledge that the material is untrue [7, 14, 17, 18]. The motives of these two groups are likely to be quite different, as is their susceptibility to interventions. For example, if an individual is sharing false material accidentally, then prompting them to focus on accuracy may well be effective. If they are doing it on purpose, already knowing it is false, then an accuracy prompt will have little effect.

It is thus important–both from the perspective of understanding why people engage with false information, and from the perspective of developing effective interventions–to ascertain what individual differences and motivational factors lead people to share false information online, both accidentally and deliberately. That is the primary goal of the series of four studies reported here.

### Individual differences

A growing body of research has investigated the possibility that personality and other individual differences impact people’s interactions with false information online. A number of studies have found different, sometimes contradictory, effects of various personality traits [1]. At this point, it appears clear that multiple individual differences account for variance in interactions with false information, but a consensus view of what traits are important has not emerged. A further complication is that different characteristics may be associated with different types of sharing false information (accidental or deliberate).

#### Accidental engagement with false material

One key narrative around why people engage with false information is that they may fail to focus on the accuracy of the material. Rather, heuristics and cognitive biases may guide interactions, with individuals being more likely to rely on intuitive rather than deliberative cognition [e.g. 9, 19]. These interactions may include sharing, and other types of engagement (such as liking) that also contribute to algorithmic propagation. Individual differences in personality and cognitive style may contribute to this process.

In particular, a lower capacity for or tendency towards reflective thought is likely to be important. Lower performance on measures of cognitive reflection, indexing a tendency to select an intuitive but incorrect rather than a considered and correct response to a question, has been associated with belief in false material [e.g. 20, 21]. While the tendency towards intuitive rather than considered decision making is usually assessed with variants of the Cognitive Reflection Test [22], other measures of decision-making style [e.g. 23] may also index this characteristic.

The personally trait of conscientiousness may also influence interactions with false material [8, 14, 24]. One reason for this is that people lower in conscientiousness are less likely to scrutinize the accuracy of material they engage with, while more conscientious people might be expected to take more care. Thus, low conscientiousness is a potential risk factor for accidental propagation of false material.

A third characteristic that might be expected to influence accidental engagement is schizotypy. Schizotypy is a set of traits reflecting tendencies towards disordered thinking. While there are different conceptualizations, schizotypy is argued to be multidimensional in nature. The positive dimension of schizotypy refers to traits that are associated with disruptions in content of thought, including cognitive-perceptual distortions, suspicion, and paranoia [25]. One of the correlates of positive schizotypy is a tendency to rely on intuitive (as opposed to deliberative) processing. This can be seen in increases in intuitive thinking, decreases in cognitive reflection [26] and the operation of information processing biases [27]. Therefore, individuals with higher levels of trait positive schizotypy may be particularly susceptible to sharing misinformation online. Notably, trait schizotypy has also been found to relate to endorsement of conspiracy beliefs [e.g. 28], which have a crossover with misinformation. Consistent with all this, research has found that higher levels of positive schizotypy were associated with greater likelihood of reporting having shared false information [17].

Thus, a number of individual differences may be associated with sharing false information inadvertently. There is evidence to suggest the same may be true of deliberate sharing.

#### Deliberate engagement with false material

It is known that some people deliberately share false information: they know the material is untrue, but choose to share it anyway [7, 14, 17, 18]. It is likely that any individual differences predisposing people towards this behavior are different from those that might increase the likelihood of inadvertent sharing. Given that deliberately sharing false information is a socially disapproved behavior, these traits are likely to have antisocial or maladaptive attributes.

One potential candidate is psychopathy, a trait typified by high impulsivity and low empathy [29]. It is known to be associated with antisocial online behaviors such as trolling, belief in conspiracy theories, and self-reports of having shared false information [17].

Another potentially important characteristic is a ‘need for chaos’ [30]. This reflects the desire of some individuals to disrupt society in order to enhance their own (currently marginalized) status. It has been shown to influence motivation to share hostile political information [31] and self-reports of deliberately sharing false information [7].

### Motivation

Alongside individual differences, it is important to consider motivations for sharing false information [e.g. 18, 32]. As noted above, it is likely that the motives of people who share misinformation without knowing it is false, and those who spread lies on purpose, are likely to differ. In the case of deliberate propagation of false material, sharers’ motives are likely to be antisocial, or centered on some benefit to the sharer. For accidental propagation, motives may vary and include prosocial objectives.

Some recent work has drawn on past research and new data to develop a framework for describing sharing motivation that is based on users’ own accounts of why they have shared material [33]. Given that people will often have shared material in good faith–thinking it is true–it is important that this applies to sharing political information in general as well as false information specifically. The framework describes six clusters of motives. Some of these are regarded as prosocial in nature and reflect an intention to ‘make things better’ (e.g. “Prosocial Activism”), while others (e.g. “Attack or manipulation of others”) are clearly antisocial.

Different motives are likely to apply to situations where people have shared false material inadvertently, or on purpose. It seems unlikely that people motivated by a desire for “Prosocial Activism” would deliberately share falsehoods. However, people motivated by a desire for “Attack or manipulation of others” might well do so.

### Aims and hypotheses

The main contribution of this project will be to identify the individual differences and motivational factors that influence deliberate and accidental sharing of false political information. It is important to consider these separately, because different psychological processes may underly each type of sharing. Findings may therefore have implications for interventions that are more or less likely to work for different categories of social media users.

Broadly, we hypothesize that individual differences associated with biases in information processing–and particularly inattention to accuracy when making decisions–will influence ‘accidental’ sharing of false information. Individual differences reflecting a lack of concern for others, in comparison to the self, will influence deliberate sharing. Inadvertent sharing of misinformation may be motivated by multiple factors, both prosocial and antisocial. However, deliberate sharing is more likely to be motivated by antisocial factors.

The paper reports a sequence of studies intended to test these broad hypotheses using different constructs and research methods. Study 1 is a cross-sectional online survey examining the extent to which several individual differences predict self-reports of sharing false information accidentally or on purpose. Study 2 extends this by also including motivational factors. Study 3 tests the role of individual differences and motivational factors as predictors of self-reported likelihood of sharing ‘real world’ examples of false information. Study 4 comprises exploratory work, using a fine-grained methodological approach, looking at whether variables previously identified as important are associated with actual sharing of false material on Twitter.

## Study 1

Study 1 was a preregistered (https://osf.io/u8m7p) cross-sectional online survey study examining the extent to which a range of variables predicted self-reports of sharing false information either accidentally or deliberately. Data, analysis code, and materials are available at https://osf.io/d84mu/. Ethical approval came from the University of Westminster Psychology Ethics Committee, approval code ETH2122-1637. Participant recruitment and data collection took place from April 27, 2022—May 18, 2022. Participants provided informed consent by selecting the appropriate option on the online questionnaire.

We reasoned that people who are more prone to accidentally share false information would have higher levels of spontaneous decision-making style, reflecting a tendency to make impulse-based decisions (Hypothesis 1); lower levels of rational decision-making style, reflecting a tendency to not double-check information sources (Hypothesis 2), lower levels of cognitive reflection, reflecting a tendency for reduced analytic thought (Hypothesis 3); lower levels of conscientiousness, reflecting a tendency to spend less time assessing information veracity (Hypothesis 4); and higher levels of Cognitive Perceptual schizotypy (a measure of positive schizotypy), reflecting a tendency towards reliance on heuristics and cognitive biases (Hypothesis 5). In addition, we reasoned that that people who were more prone to deliberately share false information would have higher levels of Need for Chaos, reflecting a desire to upset the established order (Hypothesis 6).

### Methods

#### Participants and recruitment

We initially collected data from 670 users of the Prolific participant panel, each of whom was paid the US equivalent of GB £1.25. Based on our pre-registered inclusion criteria, 56 responses were excluded: 18 had incomplete data, 12 did not pass bot detection / fraud data quality checks embedded in Qualtrics, three withheld consent at end of study, one reported age below 18, two reported using social media only about once a month, and 20 had a zero-variance pattern of questionnaire completion that suggested inauthentic responses. The analytic sample thus comprised 614 (77% women) US residents aged 18–69 (*M* = 30.48, *SD* = 12.08). To ensure variance in political affiliation, we targeted an equal number of participants who previously identified as “Democrat”, and “Republican”. Participant characteristics are shown in Table 1.

**Table 1:** Demographic characteristics and self-reported sharing of false information across studies 1–4.

	Study 1 (*N = 614)*		Study 2 (*N =* 563)		Study 3 (*N* = 627)		Study 4 (*N =* 113)	
**Gender**	***n***	**%**	***n***	**%**	***n***	**%**	***n***	**%**
Man	126	20.5	275	48.9	308	49.1	55	48.7
Woman	473	77.0	280	49.8	316	50.4	55	48.7
Non-binary	14	2.3	6	1.1	3	0.5	3	2.7
Prefer not to say	1	0.2	1	0.2	0	0	0	0
**Highest education**	***n***	**%**	***n***	**%**	***n***	**%**	***n***	**%**
Less than high school	3	0.5	6	1.1	3	0.5	0	0
High school / secondary school	76	12.4	78	13.9	96	15.3	17	15
Some college or university	215	35.0	145	25.8	164	26.2	29	25.7
College or university degree	241	39.3	244	43.4	280	44.7	44	38.9
Master’s degree	62	10.1	66	11.7	69	11	16	14.2
Doctoral degree	17	2.8	23	4.1	15	2.4	7	6.2
**Occupational status**	***n***	**%**	***n***	**%**	***n***	**%**	***n***	**%**
Employed for wages	316	51.5	351	62.5	414	66	66	58.4
Self-employed	34	5.5	79	14.1	84	13.4	16	14.2
Unemployed but looking for work	41	6.7	33	5.9	30	4.8	8	7.1
Home-maker	36	5.9	21	3.7	30	4.8	6	5.3
Student	160	26.1	45	8.0	7	1.1	7	6.2
Retired	15	2.4	21	3.7	47	7.5	6	5.3
Unable to work for health or other reasons	12	2.0	12	2.1	15	2.4	4	3.5
**Social media use**	***n***	**%**	***n***	**%**	***n***	**%**	***n***	**%**
About once every couple of weeks	4	0.7	6	1.1	10	1.6	0	0
About once a week	4	0.7	8	1.4	20	3.2	1	0.9
A few times a week	16	2.6	42	7.5	45	7.2	3	22.7
About once a day	62	10.1	96	17.1	137	21.9	12	10.6
Several times a day	528	86.0	410	73.0	415	66.2	97	98.5
**Political Ideology**	***n***	**%**	***n***	**%**	***n***	**%**	***n***	**%**
1 (Left)	119	19.4	106	18.9	1	0.2	46	40.7
2	127	20.7	103	18.3	3	0.5	22	19.5
3	58	9.4	57	10.1	10	1.6	14	12.4
4 (Center)	76	12.4	46	8.2	79	12.6	13	11.5
5	107	17.4	111	19.8	186	29.7	10	8.8
6	83	13.5	93	16.5	196	31.3	5	4.4
7 (Right)	44	7.2	46	8.2	152	24.2	3	2.7
**2020 US Presidential vote**	***n***	**%**	***n***	**%**	***n***	**%**	***n***	**%**
Biden					0	0	69	61.1
Trump					627	100	16	14.2
Other					0	0	5	4.4
Did not vote					0	0	19	16.8
Prefer not to say					0	0	4	3.5
**Ethnicity**	***n***	**%**	***n***	**%**	***n***	**%**	***n***	**%**
American Indian or Alaska native					4	0.6		
Asian					22	3.5		
Black or African American					12	1.9		
Hispanic or Latino					28	4.5		
White					553	88.2		
Other					8	1.3		
**Accidentally shared false information**	***n***	**%**	***n***	**%**	***n***	**%**	***n***	**%**
No	513	83.6	435	77.4	495	78.9	84	74.4
Yes	101	16.4	127	22.6	132	21.1	29	25.7
**Deliberately shared false information**	***n***	**%**	***n***	**%**	***n***	**%**	***n***	**%**
No	552	89.9	495	88.1	570	90.9	98	86.7
Yes	62	10.1	67	11.9	57	9.1	15	13.3
**False news amplification**	***n***	**%**	***n***	**%**	***n***	**%**	***n***	**%**
Did not share any exaggerated or false news	522	85.0	475	84.5				
Shared news that seemed accurate at the time, but you later found was made up	15	2.4	13	2.3				
Shared news that was exaggerated, and you were not aware of this	37	6.0	48	8.5				
Shared news that was exaggerated, and you were aware of this	28	4.6	16	2.8				
Shared news that you thought was made up when you shared it	12	2.0	10	1.8				

#### Procedure and materials

Data collection was carried out using Qualtrics. Participants completed background information, followed by social media measures and assessments of individual differences.

Background measures were age, gender, education, country of residency, occupational status, and political ideology, on a 1 (left) to 7 (right) scale.

Social media measures were *social media use* frequency (1, not at all, to 7, several times a day), level of *trust in political information* on social media, the extent of typical *political information sharing* on social media, and the *perceived influence of political information* encountered on social media on one’s behavior. The three latter measures were rated on a 1 (not at all) to 5 (a great deal) scale.

Accidental sharing of false information was assessed using a single question: “Have you ever shared a political news story online that you later found out was made up? (yes/no)”. Deliberate sharing of false information was assessed using a single question: “And have you ever shared a political news story online that you thought AT THE TIME was made up? (yes, no)” [cf. 17, 34]. These were the main dependent variables in our analyses. We also included another measure of misinformation amplification (sharing false information without seeking to correct it) over the past month [18]. Participants who had shared false information on purpose were asked to briefly say why; these data were used elsewhere [33].

Cognitive perceptual schizotypy was assessed using the 14-item Cognitive Perceptual subscale of the Schizotypal Personality Questionnaire—Brief Revised Updated [SPQ-BRU; 35]. An example item is: “I sometimes feel that people are talking about me.” Items were rated on a 1 (strongly disagree) to 5 (strongly agree) scale, with a higher score indicating higher levels of cognitive perceptual schizotypy. An index score was computed as the mean of ratings.

Need for chaos was evaluated using the 8-item Need for Chaos scale [31]. An example item is: “I think society should be burned to the ground”. Items were rated on a 1 (strongly agree) to 7 (strongly disagree) scale, with a higher score representing higher levels of need for chaos. A composite score was computed as the mean of ratings.

Capacity for reflective thought was evaluated using a revised version of the Cognitive Reflection Test, the 4-item CRT-2 [36]. This comprises four questions where a correct response requires overriding a seemingly clear intuitive answer (e.g., “A farmer had 15 sheep and all but 8 died. How many are left?”).

Decision-making styles were evaluated using two subscales from the General Decision-Making Style questionnaire [23]. Participants completed the 5-item Spontaneous Decision-Making style subscale (e.g., “I generally make snap decisions”), and the 5-item Rational Decision-Making style subscale (e.g., “I make decisions in a logical and systematic way”) in relation to making decisions on social media. Items were rated on a 1 (strongly disagree) to 5 (strongly agree) scale. A composite mean score was calculated for each subscale.

Conscientiousness was evaluated using the 12-item Conscientiousness subscale of the Big Five Inventory-2 [37]. An example item is: “I am someone who tends to be lazy”. Items were rated on a 1 (disagree strongly) to 5 (agree strongly) scale. A mean index score was computed. A higher score indicates higher levels of conscientiousness.

### Results

Across all studies, preregistered analyses were conducted using SPSS 29 for Mac. Descriptive statistics for the key variables are shown in Table 2. Need for Chaos scores exhibited a notable positive skew and high kurtosis, suggesting that most people scored very low on this variable with only small numbers endorsing the items.

**Table 2:** Descriptive statistics for participant characteristics, Study 1 (*N* = 614).

	*M*	*SD*	Cronbach’s *α*	Range (potential)	Range (actual)	Skew	Kurtosis
**Age**	30.49	12.08		18+	18–69	1.37	1.02
**Political ideology**	3.57	1.96		1–7	1–7	0.17	-1.30
**Trust in political information on social media**	2.55	0.85		1–7	1–5	0.01	-0.03
**Influenced by political information on social media**	2.58	1.08		1–5	1–5	0.03	-0.96
**Political information sharing**	1.96	1.07		1–5	1–5	0.97	0.09
**Cognitive Perceptual schizotypy**	2.20	0.65	0.86	1–5	1–4.57	0.46	-0.16
**Need for Chaos**	1.55	0.71	0.80	1–7	1–7	2.57	10.50
**Rational Decision-Making**	3.89	0.58	0.79	1–5	2–5	-0.26	0.15
**Spontaneous Decision-Making**	2.66	0.76	0.86	1–5	1–5	0.36	-0.01
**Conscientiousness**	3.67	0.70	0.89	1–5	1.83–5	-0.15	-0.51
**CRT-2**	2.43	1.07		0–4	0–4	-0.57	-0.29

We initially computed zero-order correlations (point biserial correlations in the case of the dichotomous outcomes) between each of our dependent variables, and our predictors and potential covariates (Table 3). We then tested which variables were associated with greater likelihood of reporting (1) sharing false information accidentally in the past, and (2) sharing false information deliberately, using two separate binary logistic regressions. In each of these analyses, the predictor variables comprised those variables about which we had hypotheses, and any other variables that had a correlation of magnitude at least *r =* .2 with the outcome variables (see Table 3 –only tendency to share political information met this threshold, and only for accidental sharing).

**Table 3:** Correlations between hypothesized predictors, covariates, and indices of sharing false information, Study 1.

	Accidental sharing	Deliberate sharing	Amplification
**Age**	.04	-.06	-.09*
**Gender (M = 1, F = 2)** ^**a**^	-.07	-.00	-.12**
**Political ideology**	-.02	.04	.06
**Political information sharing**	.20**	.19**	.13**
**Need for Chaos**	.11**	.06	.04
**Spontaneous Decision-Making**	.10*	.10*	.08*
**Rational Decision-Making**	-.07	-.09*	-.04
**CRT-2** ^**b**^	.01	.00	.05
**Conscientiousness**	-.10*	-.07	-.09*
**Cognitive Perceptual schizotypy**	.15**	.16**	.11**

*p < .05

**p < .01

^a^Men and women only, *n* = 599 for these analyses.

^b^When the sample was restricted to the 314 individuals who did not report having seen the CRT-2 before, correlations with measures of sharing remained below *r* = .2.

Hypotheses 1–5 were simultaneously tested in the first binary logistic regression, using accidental sharing of false information as the outcome variable (Table 4). Consistent with H5, Cognitive Perceptual schizotypy was associated with the likelihood of reporting having shared false information by accident, with a one-point increase in Cognitive Perceptual schizotypy being associated with a 1.53 increase in the odds of sharing. Furthermore, a one-point increase in tendency to share political information online was associated with a 1.48 increase in the odds of accidentally sharing false information.

**Table 4:** Binary logistic regression: Predictors of whether participants had previously shared political stories they subsequently discovered were false, Study 1.

	*B*	*S*.*E*.	*Wald*	*df*	*p*	*Exp(B)*	95% C.I. for *EXP(B)*
**Spontaneous Decision-Making**	0.20	0.17	1.28	1	.26	1.22	[0.87, 1.71]
**Rational Decision-Making**	-0.05	0.23	0.05	1	.83	0.95	[0.60, 1.51]
**CRT-2**	0.02	0.11	0.05	1	.83	1.02	[0.82, 1.27]
**Conscientiousness**	-0.16	0.18	0.78	1	.38	0.85	[0.60, 1.22]
**Cognitive Perceptual schizotypy**	0.42	0.18	5.76	1	.02	1.53	[1.08, 2.15]
**Political information sharing**	0.39	0.10	16.02	1	< .001	1.48	[1.22, 1.79]
**Constant**	-3.24	1.35	5.75	1	.02	0.04	

Hypothesis 6 was tested in a binary logistic regression using deliberate sharing of false information as the outcome variable (Table 5). The only predictor variable was Need for Chaos, with no other potential predictors having reached the *r* = .2 threshold for inclusion. As there were two tests of Hypothesis 6 (see below), we used a Bonferroni-corrected alpha of *p* < .025 as a threshold for significance. There was no statistically significant association between Need for Chaos scores and reports of having shared false material on purpose.

**Table 5:** Binary logistic regression: Predictors of whether participants had previously shared political stories they knew at the time were false, Study 1.

	*B*	*S*.*E*.	*Wald*	*df*	*p*	*Exp(B)*	95% C.I. for *EXP(B)*
**Need for Chaos**	0.24	0.16	2.15	1	.14	1.27	[0.93, 1.74]
**Constant**	-2.57	0.30	73.87	1	< .001	0.08	

As a pre-registered secondary test of H6, we conducted an ordinal logistic regression with Need for Chaos as the single predictor, and false information amplification [18] as the dependent variable. A Bonferroni-corrected alpha of *p* < .025 was again used as a threshold for significance given that Hypothesis 6 was tested twice. Need for Chaos did not make a statistically significant contribution to the model (*b* = 0.20, *SE* = .14, *Wald* = 1.88, *p* > .17).

At the suggestion of reviewers, we supplemented our pre-registered analysis plan and also conducted regressions including all potential predictor variables and covariates. For self-reports of accidental (Table 6) and deliberate (Table 7) sharing, age, gender and political ideology were included in the first stage of the analysis. General tendency to share political information was included at the second stage, with all other hypothesised predictors and potential covariates included in the final step. The ordinal regression examining amplification of false stories within the last month was also run with all potential predictors included (Table 8).

**Table 6:** Binary logistic regression: Predictors of whether participants had previously shared political stories they subsequently discovered were false, Study 1 (all predictors).

	*B*	*S*.*E*.	*Wald*	*df*	*p*	*Exp(B)*	95% C.I. for *EXP(B)*
**Step 1**							
**Age**	0.01	0.01	0.66	1	.42	1.01	[0.99, 1.03]
**Political Ideology (L-R)**	-0.04	0.06	0.55	1	.46	0.96	[0.85, 1.08]
**Gender (M = 1, F = 2)**	-0.45	0.26	3.04	1	.08	0.64	[0.38, 1.06]
**Constant**	-0.91	0.61	2.25	1	.13	0.40	
**Step 2**							
**Age**	0.01	0.01	0.92	1	.34	1.01	[0.99, 1.03]
**Political Ideology (L-R)**	0.02	0.06	0.12	1	.73	1.02	[0.91, 1.15]
**Gender (M = 1, F = 2)**	-0.48	0.27	3.24	1	.07	0.62	[0.37, 1.04]
**Political information sharing**	0.50	0.10	25.08	1	< .001	1.64	[1.35, 2.00]
**Constant**	-2.19	0.68	10.32	1	.00	0.11	
**Step 3**							
**Age**	0.02	0.01	3.75	1	.053	1.02	[1.00, 1.04]
**Political Ideology (L-R)**	0.05	0.07	0.66	1	.42	1.05	[0.93, 1.20]
**Gender (M = 1, F = 2)**	-0.44	0.28	2.45	1	.12	0.65	[0.38, 1.12]
**Political information sharing**	0.45	0.10	19.23	1	< .001	1.57	[1.28, 1.92]
**Need for Chaos**	0.09	0.16	0.28	1	.60	1.09	[0.79, 1.50]
**Spontaneous Decision-Making**	0.14	0.18	0.63	1	.43	1.15	[0.81, 1.63]
**Rational Decision-Making**	-0.09	0.24	0.12	1	.73	0.92	[0.57, 1.48]
**CRT-2**	0.03	0.12	0.05	1	.82	1.03	[0.82, 1.29]
**Conscientiousness**	-0.24	0.20	1.44	1	.23	0.79	[0.54, 1.16]
**Cognitive Perceptual schizotypy**	0.47	0.20	5.60	1	.02	1.61	[1.09, 2.38]
**Constant**	-3.10	1.59	3.81	1	.051	0.05	

**Table 7:** Binary logistic regression: Predictors of whether participants had previously shared political stories they knew at the time were false, Study 1 (all predictors).

	*B*	*S*.*E*.	*Wald*	*df*	*p*	*Exp(B)*	95% C.I. for *EXP(B)*
**Step 1**							
**Age**	-0.02	0.01	3.32	1	.07	0.98	[0.95, 1.00]
**Political Ideology (L-R)**	0.10	0.07	2.02	1	.16	1.11	[0.96, 1.28]
**Gender (M = 1, F = 2)**	-0.03	0.34	0.01	1	.94	0.98	[0.50, 1.89]
**Constant**	-1.82	0.81	4.99	1	.03	0.16	
**Step 2**							
**Age**	-0.02	0.01	2.88	1	.09	0.98	[0.95, 1.00]
**Political Ideology (L-R)**	0.18	0.07	6.04	1	.01	1.20	[1.04, 1.38]
**Gender (M = 1, F = 2)**	-0.05	0.35	0.02	1	.88	0.95	[0.48, 1.88]
**Political information sharing**	0.59	0.12	24.67	1	< .001	1.81	[1.43, 2.29]
**Constant**	-3.43	0.92	14.01	1	< .001	0.03	
**Step 3**							
**Age**	-0.01	0.01	0.96	1	.33	0.99	[0.96, 1.01]
**Political Ideology (L-R)**	0.22	0.08	7.51	1	.01	1.25	[1.06, 1.46]
**Gender (M = 1, F = 2)**	-0.01	0.37	0.00	1	.98	0.99	[0.48, 2.04]
**Political information sharing**	0.55	0.12	19.76	1	< .001	1.74	[1.36, 2.22]
**Need for Chaos**	0.10	0.20	0.28	1	.60	1.11	[0.75, 1.64]
**Spontaneous Decision-Making**	0.14	0.22	0.37	1	.54	1.15	[0.74, 1.77]
**Rational Decision-Making**	-0.18	0.30	0.34	1	.56	0.84	[0.47, 1.51]
**CRT-2**	-0.03	0.15	0.03	1	.85	0.97	[0.73, 1.29]
**Conscientiousness**	-0.14	0.25	0.32	1	.57	0.87	[0.53, 1.41]
**Cognitive Perceptual schizotypy**	0.53	0.24	5.02	1	.03	1.70	[1.07, 2.71]
**Constant**	-4.36	2.01	4.69	1	.03	0.01	

**Table 8:** Ordinal regression: Predictors of amplification of false stories, Study 1 (all predictors).

	*Estimate*	*Std*. *Error*	*Wald*	*df*	*Sig*.	*95% CI for Estimate*
**Age**	-0.02	0.01	3.21	1	.07	[-0.04, 0.00]
**Political Ideology (L-R)**	0.14	0.07	4.41	1	.04	[0.01, 0.27]
**Gender (M = 1, F = 2)**	-0.82	0.27	9.36	1	.00	[-1.35, -0.30]
**Political information sharing**	0.35	0.10	11.01	1	< .001	[0.14, 0.55]
**Need for Chaos**	0.03	0.17	0.03	1	.87	[-0.03, 0.36]
**Spontaneous Decision-Making**	0.10	0.18	0.33	1	.57	[-0.25, 0.45]
**Rational Decision-Making**	-0.06	0.24	0.06	1	.82	[-0.53, 0.42]
**CRT-2**	0.07	0.12	0.38	1	.54	[-0.16, 0.30]
**Conscientiousness**	-0.18	0.20	0.78	1	.38	[-0.57, 0.22]
**Cognitive Perceptual schizotypy**	0.31	0.20	2.37	1	.12	[-0.08, 0.69]

As Table 6 shows, the pattern of statistically significant predictors of accidental sharing remained the same, with only tendency to share political information and Cognitive Perceptual schizotypy being associated with self-reports of accidental sharing.

For self-reports of deliberate sharing (Table 7), the key variable being examined, Need for Chaos, remained non-significant. A number of variables not included in the previous analysis were significantly associated with self-reports of having shared information known to be false: right-wing political ideology, general tendency to share political information, and Cognitive Perceptual schizotypy.

A similar pattern of results was observed when the effect of all predictors on amplification of false stories was examined (Table 8). Again, Need for Chaos was not a significant predictor. However, right-wing political ideology, general tendency to share political information, and male gender were.

### Discussion

Of the hypotheses we put forward, only H5 –that Cognitive Perceptual schizotypy would be associated with accidental sharing of false information–was consistent with the data. Our exploratory analyses suggested that Cognitive Perceptual schizotypy was also associated with deliberate sharing of false information, as was right-wing political ideology. Right-wing ideology and male gender were also associated with amplification of false information.

Notably, other characteristics documented in the literature as being associated with sharing false information–cognitive reflection, conscientiousness and need for chaos–did not predict sharing false information when considered in the unified model alongside Cognitive Perceptual schizotypy. While there are some statistically significant correlations with these variables in Table 3, the effect sizes are very small.

One potential reason for this, which applies particularly to cognitive reflection, is that our methodology relied on self-reports of having shared false material. This relies on accurate as well as honest responding (people may well have shared false material without knowing it). Discovering that material one has shared was actually misinformation would require feedback from one’s social network, or further research and consideration (which may be particularly unlikely in the case of people with low tendencies towards reflection). Self-reports may also be unlikely to capture processes that would affect ‘in the moment’ decisions to share or not share material. We address these issues in Studies 3 and 4.

One variable about which we did not have a priori hypotheses, general tendency to share political information, emerged as the strongest correlate of sharing false information both accidentally and on purpose, and with amplification of false stories in our exploratory analysis. This is unsurprising in the case of accidental sharing (the more you share, the more likely you are to share something false by accident) but is harder to explain in the case of deliberate sharing. People’s reasons for sharing political material are likely to have a bearing on why they share false material. This is picked up in Study 2, which addresses motivation for sharing.

## Study 2

Study 2 was a preregistered (https://osf.io/5jqz9) cross-sectional online survey study examining the extent to which a range of variables predicted self-reports of sharing false information either accidentally or deliberately. It built on Study 1 by additionally considering the role of motivation, drawing on the six clusters of motivations for sharing political information (both true and false) identified by [33]: *Prosocial Activism*; *Attack or manipulation of others*; *Entertainment*; *Awareness; Political Self-expression*; and *Fighting False Information*. Data, analysis code, and materials are available at https://osf.io/d84mu/. Ethical approval came from the University of Westminster College of Liberal Arts and Sciences Research Ethics Committee, approval code ETH2223-1041. Participant recruitment and data collection took place from January 31^st^, 2023—February 9^th^, 2023. Participants provided informed consent by selecting the appropriate option on the online questionnaire.

Antisocial, disruptive motivations for sharing are potentially served by sharing knowingly false, exaggerated stories [38]. Accordingly, we hypothesize that people who share political information online to attack or manipulate others will be more likely to have deliberately shared false information online (Hypothesis 1). However, people who wish to minimize harm done by misinformation might knowingly share false information in order to counter or debunk it [39]. Therefore, we hypothesize that people motivated by a desire to fight false information will also be more likely to have deliberately shared false information (Hypothesis 2). People may also be motivated to share political material online because they believe in transparency and freedom of information. This might be the case for false material they believe is true, and potentially also information they believe to be false (because they argue that ‘there is no harm in hearing people’s opinions’). The framework advanced by [33] labelled this cluster of motives ‘Awareness’. Based on this argument that the pursuit of freedom of information online can involve sharing of information regardless of its perceived accuracy, we hypothesize that people motivated by Awareness will be more likely to report having shared false information online both deliberately (Hypothesis 3) and accidentally (Hypothesis 4). Finally, to test whether the findings of Study 1 replicate, we hypothesize that higher levels of Cognitive Perceptual schizotypy will be associated with higher levels of accidentally sharing false political information (Hypothesis 5).

### Methods

#### Participants and recruitment

We initially collected data from 718 users of the Prolific participant panel, each of whom was paid the US equivalent of GB £1.20. Based on our pre-registered inclusion criteria, 155 responses were excluded: 11 did not pass bot detection / fraud data quality checks embedded in Qualtrics, six withheld consent at end of study, four reported using social media only about once a month, and 134 had a zero-variance pattern of questionnaire completion that suggested inauthentic responses. One further participant was excluded due to a technical error with Qualtrics. The analytic sample thus comprised 562 (49.8% women) US residents aged 18–72 (*M* = 37.16, *SD* = 13.18). To ensure variance in political affiliation, we targeted an equal number of participants who previously identified as “Democrat”, and “Republican”. Participant characteristics are shown in Table 1.

#### Procedure and materials

Data collection was carried out using Qualtrics. Participants completed the same background measures and social media measures as in Study 1. Motives for sharing political information on social media were assessed using an 18-item questionnaire that provides a parsimonious set of short scales addressing six key clusters of users’ own accounts of motives for sharing both true and false political information [33]. This questionnaire was comprised of six 3-item subscales, evaluating Prosocial Activism (e.g., “to inform people”), Attack or manipulation of others (e.g., “to manipulate what people think”), Entertainment (e.g., “to be funny”), Awareness (e.g., “to balance the bias in the media”), Political Self-expression (e.g., “to express my political beliefs”), and Fighting False Information (e.g., “to point out this material is false or misleading”). Items were rated on a 1 (never for this reason) to 5 (very often for this reason) scale. We calculated a mean index score for each subscale, with a higher score representing higher levels of motive endorsement. These scores represent individuals’ motives for sharing political information in general, with no distinction made as to whether it is believed to be true or false.

Accidental sharing of false information was assessed using a single question: “Have you ever shared a political news story online that you later found out was made up?” (yes, no). Those who replied “Yes” to this question were asked to complete the questionnaire evaluating motives for sharing political information on social media a second time, with regards to the story that they had just indicated sharing and later found to be made up. The instructions requested answering for the story that comes most readily to mind if more than one such had been shared. Deliberate sharing of false information was assessed using a single question: “And have you ever shared a political news story online that you thought AT THE TIME was made up?” (yes, no). Those who replied “Yes” to this question were asked to complete the questionnaire evaluating motives for sharing political information on social media another time, with regards to the story that they had just indicated sharing and thinking at the time was made up. The instructions requested answering for the one that comes most readily to mind if more than one such story had been shared. In addition, we included a measure of false information amplification in the past month [18]. Finally, Cognitive Perceptual schizotypy was again measured with the SPQ-BRU [35].

### Results

Descriptive statistics for the key variables are shown in Table 9. We initially computed zero-order correlations (point biserial correlations in the case of the dichotomous outcomes) between each of our dependent variables, and our predictors and potential covariates (Table 10). We then used two separate binary logistic regressions to test which variables were associated with greater likelihood of reporting sharing false information deliberately (Table 11, testing hypotheses 1–3) in the past, and sharing false information accidentally (Table 12, testing hypotheses 4–5). In each of these analyses, the predictor variables comprised those variables about which we had hypotheses, and any other variables that had a correlation of magnitude at least *r =* .2 with the outcome variables (see Table 10).

**Table 9:** Descriptive statistics for participant characteristics, Study 2 (*N* = 562).

	*M*	*SD*	Cronbach’s *α*	Range (potential)	Range (actual)	Skew	Kurtosis
**Age**	37.16	13.18		18+	18–72	0.64	-0.58
**Political ideology**	3.74	2.02		1–7	1–7	0.03	-1.40
**Trust in political information on social media**	2.44	0.89		1–5	1–5	0.22	-0.23
**Influenced by political information on social media**	2.42	1.07		1–5	1–5	0.25	-0.75
**Political information sharing**	2.03	1.07		1–5	1–5	0.80	-0.30
**Prosocial activism**	3.03	1.21	.90	1–5	1–5	-0.15	-1.07
**Attack or manipulation of others**	1.31	0.62	.81	1–5	1–5	2.46	6.48
**Entertainment**	2.69	1.25	.92	1–5	1–5	0.13	-1.10
**Awareness**	2.30	1.01	.71	1–5	1–5	0.53	-0.43
**Political self-expression**	3.06	1.22	.92	1–5	1–5	-0.17	-1.03
**Fighting false information**	2.45	1.15	.87	1–5	1–5	0.34	-0.84
**Cognitive Perceptual schizotypy**	2.22	0.68	.86	1–5	1.07–4.50	0.44	-0.36

**Table 10:** Correlations between hypothesized predictors, covariates, and indices of sharing false information, Study 2.

	Accidental sharing	Deliberate sharing	Amplification
**Age**	.03	-.06	-.17**
**Gender (M = 1, F = 2)** ^**a**^	-.14**	-.19**	-.11*
**Political ideology**	.13**	.09*	.12**
**Political information sharing**	.22**	.13**	.12**
**Prosocial activism**	.15**	-.01	.05
**Attack or manipulation of others**	.20**	.25**	.28**
**Entertainment**	.21**	.15**	.09*
**Awareness**	.26**	.15**	.20**
**Political self-expression**	.17**	-.01	.06
**Fighting false information**	.20**	.15**	.09*
**Cognitive Perceptual schizotypy**	.16**	.16**	.23**

*p < .05

**p < .01

^a^Men and women only, *n* = 555 for these analyses.

**Table 11:** Binary logistic regression: Predictors of whether participants had previously shared political stories they knew at the time were false, Study 2.

	*B*	*S*.*E*.	*Wald*	*df*	*p*	*Exp(B)*	95% C.I. for *EXP(B)*
**Attack or manipulation of others**	0.72	0.18	16.37	1	< .001	2.06	[1.45, 2.92]
**Fighting false information**	0.25	0.14	3.23	1	.07	1.28	[0.98, 1.68]
**Awareness**	0.10	0.16	0.35	1	.55	1.10	[0.80, 1.51]
**Constant**	-3.94	0.44	80.90	1	< .001	0.02	

**Table 12:** Binary logistic regression: Predictors of whether participants had previously shared political stories they subsequently discovered were false, Study 2.

	*B*	*S*.*E*.	*Wald*	*df*	*p*	*Exp(B)*	95% C.I. for *EXP(B)*
**Political information sharing**	0.21	0.11	3.77	1	.052	1.24	[1.00, 1.53]
**Cognitive Perceptual schizotypy**	0.30	0.17	3.22	1	.07	1.34	[0.97, 1.86]
**Entertainment**	0.26	0.09	7.98	1	.005	1.30	[1.08, 1.56]
**Awareness**	0.22	0.14	2.62	1	.11	1.24	[0.96, 1.62]
**Fighting false information**	0.17	0.11	2.56	1	.11	1.19	[0.96, 1.47]
**Attack or manipulation of others**	0.20	0.17	1.49	1	.22	1.22	[0.89, 1.69]
**Constant**	-4.38	0.50	75.47	1	< .001	0.01	

For reported deliberate sharing of false information, only H1 was supported. A one-point increase in Attack or manipulation of others was associated with a 2.06 increase in the odds of deliberately sharing false information. Thus, Attack or manipulation of others was the only significant predictor of deliberately sharing false information when taking into account all other hypothesized predictors (Table 11).

Only sharing motivated by Entertainment was statistically significantly associated with self-reports of having shared material later found to be false, with a one-point increase being associated with a 1.30 increase in the odds of sharing (Table 12).

As in Study 1 we also conducted additional versions of these analyses, summarized in Tables 13 and 14, which included all plausible potential predictors of the outcome variables. For deliberate sharing, inclusion of additional variables changed the picture somewhat. The Attack motive for sharing (just) became non-significant, while male gender, general tendency to share political information, lower motivation to share for reasons of Political Self-expression, sharing to fight false information, and Cognitive Perceptual schizotypy were all associated with reports of having shared stories known at the time to be false. For accidental sharing, the Entertainment motivation remained significant, while male gender, right-wing political ideology, and Cognitive Perceptual schizotypy were significantly associated with reports of having shared stories later discovered to be false.

**Table 13:** Binary logistic regression: Predictors of whether participants had previously shared political stories they thought at the time were false, Study 2 (all predictors).

	*B*	*S*.*E*.	*Wald*	*df*	*p*	*Exp(B)*	95% C.I. for *Exp(B)*
**Step 1**							
**Age**	-0.01	0.01	0.81	1	.37	0.99	[0.97, 1.01]
**Gender (M = 1, F = 2)**	-1.17	0.30	15.39	1	< .001	0.31	[0.17, 0.56]
**Political ideology**	0.12	0.07	3.10	1	.08	1.13	[0.99, 1.30]
**Constant**	-1.67	0.49	11.83	1	< .001	0.19	
**Step 2**							
**Age**	-0.01	0.01	1.27	1	.26	0.99	[0.97, 1.01]
**Gender (M = 1, F = 2)**	-1.13	0.30	14.10	1	< .001	0.32	[0.18, 0.58]
**Political ideology**	0.13	0.07	3.60	1	.06	1.14	[1.00, 1.31]
**Political information sharing**	0.38	0.12	10.04	1	.002	1.46	[1.15, 1.84]
**Constant**	-2.46	0.55	19.67	1	< .001	0.09	
**Step 3**							
**Age**	-0.01	0.01	0.86	1	.35	0.99	[0.97, 1.01]
**Gender (M = 1, F = 2)**	-0.96	0.32	9.21	1	.002	0.38	[0.21, 0.71]
**Political ideology**	0.08	0.08	1.03	1	.31	1.09	[0.93, 1.27]
**Political information sharing**	0.34	0.16	4.44	1	.04	1.40	[1.02, 1.92]
**Prosocial activism**	-0.25	0.17	2.01	1	.16	0.78	[0.56, 1.10]
**Attack or manipulation of others**	0.38	0.20	3.80	1	.051	1.47	[1.00, 2.15]
**Entertainment**	0.18	0.13	2.04	1	.15	1.20	[0.93, 1.54]
**Awareness**	0.05	0.21	0.06	1	.81	1.05	[0.69, 1.61]
**Political self-expression**	-0.43	0.19	5.15	1	.02	0.65	[0.45, 0.94]
**Fighting false information**	0.52	0.17	9.42	1	.002	1.68	[1.21, 2.33]
**Cognitive Perceptual schizotypy**	0.47	0.22	4.53	1	.03	1.60	[1.04, 2.47]
**Constant**	-3.92	0.84	21.53	1	< .001	0.02	

**Table 14:** Binary logistic regression: Predictors of whether participants had previously shared political stories they subsequently discovered were false, Study 2 (all predictors).

	*B*	*S*.*E*.	*Wald*	*df*	*p*	*Exp(B)*	95% C.I. for *Exp(B)*
**Step 1**							
**Age**	0.01	0.01	1.14	1	.29	1.01	[0.99, 1.02]
**Gender (M = 1, F = 2)**	-0.66	0.21	9.40	1	.002	0.52	[0.34, 0.79]
**Political ideology**	0.16	0.05	8.67	1	.003	1.17	[1.05, 1.30]
**Constant**	-1.88	0.37	25.41	1	< .001	0.15	
**Step 2**							
**Age**	0.01	0.01	0.52	1	.47	1.01	[0.99, 1.02]
**Gender (M = 1, F = 2)**	-0.60	0.22	7.51	1	.006	0.55	[0.36, 0.84]
**Political ideology**	0.18	0.06	10.44	1	.001	1.19	[1.07, 1.33]
**Political information sharing**	0.50	0.10	26.66	1	< .001	1.64	[1.36, 1.98]
**Constant**	-2.96	0.45	44.04	1	< .001	0.05	
**Step 3**							
**Age**	0.01	0.01	0.31	1	.58	1.01	[0.99, 1.02]
**Gender (M = 1, F = 2)**	-0.52	0.23	5.10	1	.02	0.60	[0.38, 0.93]
**Political ideology**	0.16	0.06	7.11	1	.008	1.17	[1.04, 1.32]
**Political information sharing**	0.22	0.12	3.38	1	.07	1.25	[0.99, 1.59]
**Prosocial activism**	0.03	0.13	0.07	1	.79	1.04	[0.80, 1.34]
**Attack or manipulation of others**	0.14	0.17	0.67	1	.41	1.15	[0.82, 1.61]
**Entertainment**	0.25	0.10	6.47	1	.01	1.28	[1.06, 1.55]
**Awareness**	0.11	0.15	0.50	1	.48	1.12	[0.83, 1.51]
**Political self-expression**	0.09	0.14	0.41	1	.52	1.09	[0.84, 1.42]
**Fighting false information**	0.17	0.12	1.89	1	.17	1.18	[0.93, 1.50]
**Cognitive Perceptual schizotypy**	0.37	0.17	4.52	1	.03	1.44	[1.03, 2.03]
**Constant**	-5.13	0.71	52.17	1	< .001	0.01	

We also conducted an exploratory ordinal logistic regression, examining which potential predictor variables were associated with self-reports of having amplified false stories without trying to correct them in the past month (Table 15). This showed that people who were more likely to have amplified false stories were younger, and were motivated to share political information in order to Attack or manipulate others. They also scored higher on Cognitive Perceptual schizotypy.

**Table 15:** Ordinal regression: Predictors of amplification of false stories, Study 2 (all predictors).

	*Estimate*	*Std*. *Error*	*Wald*	*df*	*p*	*95% C*.*I*. *for Estimate*
**Age**	-0.04	0.01	12.88	1	< .001	[-0.06, -0.02]
**Gender (M = 1, F = 2)**	-0.27	0.26	1.06	1	.30	[-0.78, 0.24]
**Political ideology**	0.12	0.07	3.22	1	.07	[-0.01, 0.26]
**Political information sharing**	0.15	0.14	1.20	1	.27	[-0.12, 0.42]
**Prosocial activism**	-0.09	0.15	0.38	1	.54	[-0.39, 0.21]
**Attack or manipulation of others**	0.53	0.17	9.19	1	.002	[0.19, 0.87]
**Entertainment**	0.02	0.11	0.04	1	.85	[-0.20, 0.24]
**Awareness**	0.24	0.18	1.79	1	.18	[-0.11, 0.59]
**Political self-expression**	-0.06	0.16	0.13	1	.72	[-0.37, 0.25]
**Fighting false information**	0.15	0.14	1.13	1	.29	[-0.13, 0.42]
**Cognitive Perceptual schizotypy**	0.50	0.19	6.81	1	.009	[0.13, 0.88]

Finally, we considered the motivations for sharing those specific stories indicated by those individuals who had reported sharing false information accidentally or deliberately. Mean scores on each motivation for each type of sharing are shown in Table 16. In terms of highest mean scores, the most important motivation for sharing information that was later found to be false was Prosocial Activism, followed by Political Self-expression. The most important motivation for deliberately sharing false information was Entertainment, followed by Fighting False Information.

**Table 16:** Ratings of reasons for sharing specific false stories, accidentally and deliberately, Study 2.

	Accidental (*n* = 127)		Deliberate (*n* = 67)	
	***M***	***SD***	***M***	***SD***
**Prosocial activism**	3.32	1.16	2.86	1.28
**Attack or manipulation of others**	1.54	0.87	1.80	1.03
**Entertainment**	2.75	1.27	3.43	1.23
**Awareness**	2.47	1.15	2.59	1.09
**Political self-expression**	3.21	1.10	2.78	1.12
**Fighting false information**	2.53	1.23	3.02	1.30

### Discussion

Of the hypotheses we advanced, the data were only consistent with H1: people who tended to share political information for the purpose of attacking or manipulating others, were more likely to report having shared false information on purpose (though in our exploratory analysis including more predictors, it became non-significant at *p =* .051). The Attack motive was also associated with the conceptually related measure of false information amplification. Notably however, when people were asked about their reasons for sharing a specific story they knew was false, the Attack motive was assigned the lowest rating on average.

Hypothesis 2, that people motivated by a desire to fight false information would be more likely to report having deliberately shared it, was not supported by our initial analysis. However, our exploratory analysis including more predictors, was consistent with the prediction, indicating that the effect might operate when other variables are controlled for.

We had expected that Cognitive Perceptual schizotypy would predict reported accidental sharing. It was significantly correlated with accidental sharing, though with a small effect size (Table 10). When considered alongside other variables in the regression analysis (Table 12), it did not reach statistical significance (*p* = .07), though in our exploratory analysis including more variables (Table 14) it was significant, at *p* = .03. However, it did statistically significantly predict scores on the amplification measure (which is closer to deliberate than accidental sharing, indexing the number of times in the past month participants had shared false material without seeking to correct it; Table 15), and in our extended analysis it also predicted self-reports of deliberately sharing false stories (Table 13). Overall, this reinforces findings from Study 1 (and past research) that Cognitive Perceptual schizotypy is relevant to sharing false information, but raises questions about the effect size, mechanism, and how it interacts with other predictors.

We hypothesized that the ‘Awareness’ motivation would predict both deliberate and accidental sharing. However, it did not emerge as a significant predictor in either regression, despite the fact it had the largest correlation with accidental sharing (Table 10). This raises the possibility that its effect is fully mediated by another variable included in the analysis.

An unanticipated finding was that the Entertainment motive predicted accidental sharing. It is easy to see how some material people shared because they thought it was funny could turn out to be untrue (perhaps particularly in the case of humorous political memes). Other additional findings emerged from our exploratory analyses (Tables 13–15). Male gender was associated with self-reports of both deliberate and accidental sharing. General tendency to share political information, and lower levels of motivation to share for reasons of Political Self-expression, were also associated with self-reports of deliberate sharing. Right-wing political ideology was associated with reports of accidental sharing. Younger age was associated with amplification of false stories in the past month.

Finally, it is worth noting that different motivational variables predicted self-reports of deliberate and accidental sharing. Respondents also apportioned most weight to different motivations when probed as to their reasons for sharing specific false stories either accidentally or deliberately. This reinforces the notion that that we need to consider different motivations for different types of sharing.

As noted previously, reliance on self-report methodology can potentially introduce response biases. Variables that influence ‘in the moment’ decisions about sharing might not influence self-reports of historical behavior in the same way. We sought to address this in Study 3.

## Study 3

Studies 1 and 2 documented associations of individual differences and motivational variables with self-reports of having shared false information. Study 3 set out to test the same hypotheses, using an alternative scenario-based methodology used in previous misinformation research [34].

We hypothesized that there would be a positive association between an individual’s level of positive schizotypy and the extent to which they rate themselves as likely to spread examples of false stories (H1), and planned to test this using two different measures of positive schizotypy. We further hypothesized that there would be a positive association between an individual’s motivation to share political information online in order to attack or manipulate others, and the extent to which they rate themselves as likely to spread examples of false stories (H2).

Study 3 was a preregistered (https://osf.io/sfu52) cross-sectional online survey study examining the extent to which a range of variables predicted participants ratings of how likely they would be to share a selection of false stories. Data, analysis code, and materials are available at https://osf.io/d84mu/. Ethical approval came from the University of Westminster College of Liberal Arts and Sciences Research Ethics Committee, approval code ETH2223-2858. Participant recruitment and data collection took place from July 7, 2023—July 15, 2023. Participants provided informed consent by selecting the appropriate option on the online questionnaire.

### Methods

#### Participants and recruitment

Participants were members of the Prolific research panel, each of whom was paid the US equivalent of GBP £2.40. Prolific’s embedded pre-screeners were used to recruit a balanced sample of male and female participants who were resident in the USA; used social media at least once a month; and had voted for Donald Trump in the 2020 US presidential election. Only Trump voters were recruited because the stimuli they would be asked to rate were Republican-favoring, not because we had any specific expectations about members of that group. We initially collected data from 700 respondents. Based on our pre-registered inclusion criteria, 73 responses were excluded: 26 did not pass bot detection / fraud data quality checks embedded in Qualtrics, 18 reported using social media ’not at all’ or only ’once a month’, three withheld consent at end of study, and two had a zero-variance pattern of questionnaire completion that suggested inauthentic responses. Additionally, 24 who did not report voting for Trump in 2020, and thus violated our inclusion criterion, were removed. The analytic sample thus comprised 627 respondents. Demographic characteristics of participants are shown in Table 1. The sample was largely white, with post-secondary education, and paid employment. The demographic profile is largely a result of the study’s inclusion criteria.

#### Procedure and materials

Data collection was carried out using Qualtrics. Participants completed the same background measures and social media measures as in Study 1. In addition to those constructs about which we had hypotheses, participants completed a battery of other measures for use in exploratory analyses.

Participants first completed a series of demographic and political questions. They then completed the main dependent measure, self-rated likelihood of sharing false information online, which was derived from a procedure described by [34]. Participants were shown, in randomized order, five true and five false political stories. Each comprised a headline with an accompanying image. The stimuli were drawn from a library of headlines previously rated for partisanship [40]. All were right-wing in their political orientation, with scores above the midpoint of a 6-point partisanship rating. We chose to use right-wing headlines due to their greater availability in the database than headlines with a left-wing orientation. The mean partisanship ratings for the sets of true and false items we selected were virtually identical. Example stimuli were “Liberals at UT Austin Want Masculinity Designated As A Mental Illness” (false) and “USPS flashback: Obama administration removed thousands of mailboxes” (true). For each of the ten stories, participants indicated whether they would consider sharing the story online, for example through Facebook or Twitter (response options no, maybe, yes). A total false-information-sharing score was computed by summing responses across the five false stories, with ’yes’ scored as 2, ’maybe’ scored as 1, ’no’ scored as zero. Having rated their willingness to share all ten stories, participants were then asked to rate the likelihood that each of the stories were true. Several other variables can be derived from the combination of these two tasks, including accidental sharing of false information (willingness to share stories that were false but which participants did not indicate were likely to be untrue); deliberate sharing of false information (willingness to share stories that were false and which participants indicated they thought were likely to be untrue); and accuracy of judgements as to whether stories were true or false (truth detection), according to the methods described in [34].

Participants then answered a series of questions about social media and their interaction with political information online, including sharing of false political information (both knowingly and accidentally). They also answered a negatively worded item ’As far as I am aware, I have never shared a political news story online that was made up, even by accident’ (t/f) which was used as a test for yea-saying biases in responses. Participants then completed, in turn, the 18-item measure of reasons for sharing political information [33]; the Cognitive Perceptual Schizotypy scale of the Schizotypal Personality Questionnaire (SPQ-BRU) [35]; and the Positive Schizotypy scale of the Multidimensional Schizotypy Scale-Brief (MSS-B) [41]. This contains 13 items, such as “I believe that there are secret signs in the world if you just know how to look for them”, answered in a true/false format. Participants then completed the Psychopathy scale of the Short Dark Triad (SD3) [42]; the CRT-2 measure of cognitive reflection [36]; and the Rational and Spontaneous scales of the General Decision-Making Style questionnaire (GDMS) [23]. After this, participants answered an attention check item to enable checks on data quality. This was placed after the CRT-2, decision making measures, and dependent measures to avoid biasing those by inducing a more reflective response set. Participants then completed the Conscientiousness and Agreeableness scales of the Big Five Inventory 2 (BFI-2) [37]; and the Fun humor style scale of the Comic Style Markers (CSM) [43].

### Results

Descriptive statistics for the key variables are shown in Table 17. We noted a non-normal distribution for the ‘Attack or manipulation’ motive scale, with a potential reason for this being reluctance to endorse the items (or its genuine rarity as a motive for sharing political information).

**Table 17:** Descriptive statistics for participant characteristics, Study 3 (*N* = 627).

	*M*	*SD*	Cronbach’s *α*	Range (potential)	Range (actual)	Skew	Kurtosis
**Age**	44.79	13.53		18+	20–80	0.22	-0.95
**Political ideology**	5.62	1.08		1–7	1–7	-0.50	0.07
**Trust in political information on social media**	2.24	0.87		1–7	1–5	0.18	-0.44
**Influenced by political information on social media**	2.04	1.06		1–5	1–5	0.65	-0.63
**Political information sharing**	1.91	0.96		1–5	1–5	0.97	0.45
**Accuracy of truth rating for true and false stories (truth detection)**	22.50	3.97		0–40	9–33	-0.15	0.09
**Likely overall false information sharing**	2.77	2.78		0–10	0–10	0.81	-0.37
**Likely true information sharing**	2.94	2.61		0–10	0–10	0.61	-.54
**Likely known false stories they would share**	0.53	0.83		0–5	0–4	1.67	2.63
**Likely unknown false stories they would share**	1.36	1.44		0–5	0–5	0.91	-0.08
**Prosocial activism**	3.04	1.38	0.94	1–5	1–5	-0.24	-1.27
**Attack or manipulation of others**	1.24	0.60	0.78	1–5	1–5	3.25	11.58
**Entertainment**	2.53	1.30	0.92	1–5	1–5	0.24	-1.27
**Awareness**	2.39	1.14	0.75	1–5	1–5	0.32	-1.01
**Political self-expression**	2.93	1.38	0.94	1–5	1–5	-0.08	-1.33
**Fighting false information**	2.14	1.20	0.90	1–5	1–5	0.76	-0.55
**Cognitive Perceptual Schizotypy**	1.78	0.69	0.90	1–5	1–4.86	1.05	1.05
**MSS-B Positive Schizotypy**	1.30	2.25	0.84	0–13	0–13	2.71	8.91
**Psychopathy**	16.57	5.51	0.78	9–45	9–36	0.82	0.46
**Rational Decision-Making**	4.10	0.64	0.85	1–5	1.6–5	-0.76	1.06
**Spontaneous Decision-Making**	2.33	0.84	0.89	1–5	1–5	0.64	0.07
**Conscientiousness**	4.04	0.74	0.92	1–5	1.33–5	-0.69	0.00
**Agreeableness**	3.93	0.66	0.88	1–5	1.92–5	-0.47	-0.20
**Aggression**	2.16	0.68	0.84	1–5	1–4.33	0.72	0.08
**Fun humor style**	4.02	1.55	0.93	1–7	1–7	-0.30	-0.77
**CRT-2**	2.58	1.13	0.64	0–4	0–4	-0.70	-0.25

Hypothesis 1, that positive schizotypy would be correlated with false information sharing, was tested with both schizotypy scales. SPQ-BRU Cognitive Perceptual schizotypy correlated positively and significantly with false information sharing (*r* = .10, *p* = .01, *n =* 627, alpha = .025 due to Bonferroni correction for multiple hypothesis testing), as did MSS-B Positive Schizotypy (*r =* .143, *p* < .001, *n* = 627, alpha = .025) though with small effect sizes.

Hypothesis 2, that tendency to share political information for the purpose of attacking or manipulating others would be correlated with false information sharing, was also consistent with the data (*r* = .23, *p* < .001, *n* = 627). Given concerns about the distributional properties of this motivational variable, we also computed a Spearman correlation for this relationship: this was also positive and statistically significant (*rho =* .23, *p* < .001, *n* = 627).

We then conducted a series of exploratory analyses. Correlations between measured variables, likelihood of sharing false information and likelihood of sharing stories known to be false, are shown in Table 18 (for comparability with Study 2, Table 18 also includes point-biserial correlations with self-reports of having shared false stories deliberately or accidentally). Given the large sample size, and large number of correlations and corresponding risk of false-positive errors, we considered effect sizes (absolute magnitude of correlations) rather than statistical significance and used a threshold of *r =* .2 for inclusion of predictors we did not have hypotheses about in further analyses.

**Table 18:** Correlations between hypothesized predictors, covariates, and indices of sharing false information, Study 3.

	Likely overall false information sharing	Likely known false stories they would share	Self-reported Accidental sharing	Self-reported Deliberate sharing	Accuracy of truth rating
**Self-reported accidental sharing**	.29***	.19***		.33***	-.04
**Self-reported deliberate sharing**	.20***	.17***	.33***		-.04
**Accuracy of truth rating**	-.27***	.17***	-.04	-.04	
**Age**	.07	.01	-.01	-.14***	-.02
**Political ideology**	.21***	.06	.07	.00	-.19***
**Political information sharing**	.54***	.27***	.38***	.25***	-.09*
**Cognitive Perceptual Schizotypy**	.10***	.08*	.13**	.19***	-.04
**MSS-B Positive Schizotypy**	.14***	.07	.14***	.15***	-.02
**Prosocial activism**	.49***	.24***	.32***	.09*	-.07
**Attack or manipulation of others**	.23***	.13**	.21***	.40***	-.08
**Entertainment**	.28***	.21***	.27***	.26***	-.02
**Awareness**	.53***	.28***	.37***	.20***	-.09*
**Political self-expression**	.52***	.25***	.31**	.13***	-.04
**Fighting false information**	.41***	.19***	.28**	.25***	-.07
**Psychopathy**	.10*	.03	.15**	.24***	-.06
**Rational Decision-Making**	.02	-.01	-.14**	-.08*	.06
**Spontaneous Decision-Making**	.09*	.00	.14**	.18***	-.06
**Conscientiousness**	-.05	.00	-.17**	-.13**	.08*
**Agreeableness**	.02	.01	-.11**	-.17***	.10*
**Aggression**	.17***	.06	.19***	.21***	-.07
**Fun humor style**	.16***	.09*	.08*	.10*	-.02
**CRT-2**	-.16**	-.06	-.04	-.04	.04
**Gender (M = 1, F = 2)** ^**a**^	-.13**	-.04	-.05	-.12**	.03

*p < .05

** *p* < .01

***p < .001

^a^Men and women only, *n* = 624 for these analyses.

Political ideology, tendency to share political information, and all of the motivation variables correlated *r >* .2 with overall likelihood of sharing false stories. These were entered in a multiple regression analysis as predictors of that variable, alongside the MSS-B Positive Schizotypy score (used because it had a larger effect size than the SPQ-BRU Cognitive Perceptual schizotypy scale). As shown in Table 19, only right-leaning political ideology, tendency to share political information in general, and sharing for reasons of Awareness predicted overall likelihood of sharing false stories.

**Table 19:** Predictors of overall likelihood of sharing false stories, Study 3.

	*B*	*SE*	*β*	*t*	*p*	Tolerance	VIF
**(Constant)**	-2.78	0.52		-5.30	< .001		
**MSS-B Positive Schizotypy**	0.01	0.04	0.01	0.14	.89	0.89	1.13
**Political ideology**	0.26	0.09	0.10	3.12	.002	0.93	1.08
**Political information sharing**	0.81	0.13	0.28	6.32	< .001	0.51	1.98
**Prosocial activism**	0.12	0.11	0.06	1.05	.29	0.33	3.08
**Attack or manipulation of others**	0.00	0.17	0.00	0.01	1.00	0.79	1.26
**Entertainment**	0.06	0.08	0.03	0.82	.41	0.74	1.35
**Awareness**	0.49	0.13	0.20	3.86	< .001	0.37	2.73
**Political self-expression**	0.17	0.12	0.08	1.43	.16	0.30	3.37
**Fighting false information**	0.16	0.10	0.07	1.63	.10	0.57	1.76
***R*** ^***2***^		0.39					
**Adjusted *R*** ^***2***^		0.38					
***F***		43.38			< .001		

Fewer variables correlated *r*>.2 with likelihood of sharing stories known to be false (Table 18): tendency to share political information, and the motivational variables Prosocial Activism, Entertainment, Awareness, and Political Self-expression. These were entered in a multiple regression analysis as predictors of that variable, alongside the MSS-B positive schizotypy score, and the Attack motivation variable. As shown in Table 20, only general tendency to share political information, sharing for Entertainment, and sharing for Awareness predicted likelihood of deliberately sharing false stories.

**Table 20:** Predictors of likelihood of sharing known false stories, Study 3.

	*B*	*SE*	*β*	*t*	*p*	Tolerance	VIF
**(Constant)**	-0.19	0.19		-1.01	.31		
**MSS-B Positive Schizotypy**	-0.01	0.02	-0.01	-0.34	.73	0.89	1.13
**Political ideology**	0.01	0.03	0.01	0.20	.84	0.93	1.08
**Political information sharing**	0.13	0.05	0.15	2.82	.01	0.51	1.98
**Prosocial activism**	0.02	0.04	0.03	0.39	.70	0.33	3.00
**Attack or manipulation of others**	0.00	0.06	0.00	0.03	.97	0.81	1.23
**Entertainment**	0.06	0.03	0.10	2.21	.03	0.76	1.32
**Awareness**	0.10	0.05	0.14	2.29	.02	0.38	2.60
**Political self-expression**	0.00	0.04	-0.01	-0.08	.94	0.30	3.35
***R*** ^***2***^		0.32					
**Adjusted *R*** ^***2***^		0.11					
***F***		9.08			< .001		

Again, we also conducted versions of these analyses with all potential predictors, summarized in Tables 21 and 22. In addition to right-leaning political ideology, tendency to share political information, and sharing motivated by raising Awareness; the overall willingness to share false stories was also predicted by older age, male gender, and lower CRT-2 score.

**Table 21:** Predictors of overall likelihood of sharing false stories, Study 3. (all predictors).

	*B*	*SE*	*β*	*t*	*p*	Tolerance	VIF
**Model 1**							
**(Constant)**	0.38	0.70		0.55	.59		
**Age**	0.01	0.01	0.06	1.50	.14	0.95	1.05
**Gender (M-1, f = 2)**	-0.79	0.22	-0.14	-3.59	< .001	0.97	1.03
**Political Ideology**	0.54	0.10	0.21	5.27	< .001	0.98	1.02
**Model 2**							
**(Constant)**	-1.31	0.61		-2.15	.03		
**Age**	0.01	0.01	0.04	1.18	.24	0.95	1.05
**Gender (M-1, f = 2)**	-0.53	0.19	-0.09	-2.79	.01	0.96	1.04
**Political Ideology**	0.30	0.09	0.11	3.34	< .001	0.95	1.06
**Political information sharing**	1.48	0.10	0.51	15.05	< .001	0.96	1.05
**Model 3**							
**(Constant)**	-2.38	1.52		-1.56	.12		
**Age**	0.02	0.01	0.08	2.28	.02	0.89	1.13
**Gender (M-1, f = 2)**	-0.56	0.19	-0.10	-2.88	.004	0.80	1.25
**Political Ideology**	0.25	0.09	0.10	2.96	.003	0.89	1.12
**Political information sharing**	0.74	0.13	0.25	5.65	< .001	0.48	2.07
**MSS-B Positive Schizotypy**	0.00	0.04	0.00	-0.05	.96	0.78	1.29
**Prosocial activism**	0.14	0.11	0.07	1.26	.21	0.31	3.26
**Attack or manipulation of others**	-0.01	0.17	0.00	-0.07	.94	0.73	1.36
**Entertainment**	0.05	0.08	0.02	0.58	.57	0.69	1.46
**Awareness**	0.47	0.13	0.19	3.69	< .001	0.35	2.83
**Political self-expression**	0.20	0.12	0.10	1.68	.09	0.29	3.46
**Fighting false information**	0.10	0.10	0.04	1.06	.29	0.56	1.80
**Psychopathy**	-0.04	0.03	-0.08	-1.55	.12	0.41	2.45
**Rational Decision-Making**	-0.11	0.18	-0.02	-0.58	.57	0.56	1.80
**Spontaneous Decision-Making**	0.09	0.14	0.03	0.66	.51	0.54	1.86
**Conscientiousness**	-0.05	0.15	-0.01	-0.36	.72	0.61	1.63
**Agreeableness**	0.18	0.19	0.04	0.94	.35	0.47	2.11
**Aggression**	0.22	0.21	0.05	1.06	.29	0.37	2.71
**Fun humor style**	0.09	0.06	0.05	1.47	.14	0.81	1.24
**CRT-2**	-0.19	0.08	-0.08	-2.36	.02	0.90	1.11
***R***^***2***^ ***(Model 3)***		.41					
**Adjusted *R***^***2***^ ***(Model 3)***		.40					
***F (Model 3)***		22.39			< .001		

**Table 22:** Predictors of likelihood of sharing known false stories, Study 3. (all predictors).

	*B*	*SE*	*β*	*t*	*p*	Tolerance	VIF
**Model 1**							
**(Constant)**	0.34	0.22		1.57	0.12		
**Age**	0.00	0.00	0.01	0.25	0.80	0.95	1.05
**Gender (M-1, f = 2)**	-0.07	0.07	-0.04	-1.05	0.29	0.97	1.03
**Political Ideology**	0.05	0.03	0.06	1.51	0.13	0.98	1.02
**Model 2**							
**(Constant)**	0.07	0.21		0.35	0.73		
**Age**	0.00	0.00	0.00	0.01	1.00	0.95	1.05
**Gender (M-1, f = 2)**	-0.03	0.07	-0.02	-0.46	0.65	0.96	1.04
**Political Ideology**	0.01	0.03	0.01	0.32	0.75	0.95	1.06
**Political information sharing**	0.23	0.03	0.27	6.75	< .001	0.96	1.05
**Model 3**							
**(Constant)**	0.25	0.56		0.45	0.66		
**Age**	0.00	0.00	0.01	0.34	0.73	0.89	1.13
**Gender (M-1, f = 2)**	-0.03	0.07	-0.02	-0.41	0.68	0.80	1.25
**Political Ideology**	0.00	0.03	0.00	0.06	0.95	0.89	1.12
**Political information sharing**	0.12	0.05	0.14	2.52	0.01	0.48	2.07
**MSS-B Positive Schizotypy**	0.00	0.02	0.00	0.10	0.93	0.78	1.29
**Prosocial activism**	0.01	0.04	0.02	0.30	0.77	0.31	3.26
**Attack or manipulation of others**	0.02	0.06	0.02	0.35	0.73	0.73	1.36
**Entertainment**	0.07	0.03	0.10	2.23	0.03	0.69	1.46
**Awareness**	0.11	0.05	0.15	2.36	0.02	0.35	2.83
**Political self-expression**	0.01	0.04	0.01	0.19	0.85	0.29	3.46
**Fighting false information**	-0.01	0.04	-0.02	-0.36	0.72	0.56	1.80
**Psychopathy**	-0.01	0.01	-0.07	-1.13	0.26	0.41	2.45
**Rational Decision-Making**	-0.09	0.07	-0.07	-1.29	0.20	0.56	1.80
**Spontaneous Decision-Making**	-0.04	0.05	-0.04	-0.75	0.46	0.54	1.86
**Conscientiousness**	0.05	0.06	0.04	0.88	0.38	0.61	1.63
**Agreeableness**	-0.02	0.07	-0.01	-0.23	0.82	0.47	2.11
**Aggression**	-0.01	0.08	-0.01	-0.12	0.91	0.37	2.71
**Fun humor style**	0.02	0.02	0.04	0.98	0.33	0.81	1.24
**CRT-2**	-0.02	0.03	-0.02	-0.56	0.58	0.90	1.11
***R***^***2***^ ***(Model 3)***		.11					
**Adjusted *R***^***2***^ ***(Model 3)***		.09					
***F (Model 3)***		4.07			< .001		

For likelihood of sharing stories known to be false, as in the earlier analysis (Table 20), the extended analysis (Table 22) indicates that only general tendency to share political information, sharing for Entertainment, and sharing for Awareness predicted willingness to share stories known to be false.

Finally, we conducted another exploratory regression, examining predictors of likelihood of sharing true stories (Table 23). As for false stories, older age, general tendency to share political information, sharing for reasons of Awareness, and lower CRT-2 score were associated with likelihood of sharing. The pattern however differed from false stories, in that higher Agreeableness also predicted likelihood of sharing true stories, while gender and political ideology did not.

**Table 23:** Predictors of overall likelihood of sharing true stories, Study 3 (all predictors).

	*B*	*SE*	*β*	*t*	*p*	Tolerance	VIF
**Model 1**							
**(Constant)**	0.82	0.67		1.24	.22		
**Age**	0.02	0.01	0.08	1.96	.050	0.95	1.05
**Gender (M = 1, F = 2)**	-0.41	0.21	-0.08	-1.97	.049	0.97	1.03
**Political Ideology**	0.36	0.10	0.15	3.76	< .001	0.98	1.02
**Model 2**							
**(Constant)**	-0.75	0.58		-1.29	.20		
**Age**	0.01	0.01	0.06	1.72	.09	0.95	1.05
**Gender (M-1, f = 2)**	-0.17	0.18	-0.03	-0.93	.35	0.96	1.04
**Political Ideology**	0.14	0.09	0.06	1.65	.10	0.95	1.06
**Political information sharing**	1.38	0.09	0.51	14.60	< .001	0.96	1.05
**Model 3**							
**(Constant)**	-2.57	1.42		-1.81	.07		
**Age**	0.02	0.01	0.08	2.43	.02	0.89	1.13
**Gender (M-1, f = 2)**	-0.23	0.18	-0.04	-1.24	.22	0.80	1.25
**Political Ideology**	0.11	0.08	0.04	1.32	.19	0.89	1.12
**Political information sharing**	0.56	0.12	0.21	4.64	< .001	0.48	2.07
**MSS-B Positive Schizotypy**	0.02	0.04	0.02	0.49	.62	0.78	1.29
**Prosocial activism**	0.19	0.11	0.10	1.78	.08	0.31	3.26
**Attack or manipulation of others**	-0.12	0.16	-0.03	-0.74	.46	0.73	1.36
**Entertainment**	-0.02	0.08	-0.01	-0.30	.77	0.69	1.46
**Awareness**	0.54	0.12	0.24	4.49	< .001	0.35	2.83
**Political self-expression**	0.19	0.11	0.10	1.73	.08	0.29	3.46
**Fighting false information**	0.15	0.09	0.07	1.63	.10	0.56	1.80
**Psychopathy**	0.02	0.02	0.04	0.74	.46	0.41	2.45
**Rational Decision-Making**	-0.33	0.17	-0.08	-1.96	.051	0.56	1.80
**Spontaneous Decision-Making**	-0.11	0.13	-0.04	-0.84	.40	0.54	1.86
**Conscientiousness**	0.16	0.14	0.05	1.15	.25	0.61	1.63
**Agreeableness**	0.49	0.18	0.12	2.70	.007	0.47	2.11
**Aggression**	-0.01	0.20	0.00	-0.04	.97	0.37	2.71
**Fun humor style**	0.07	0.06	0.04	1.12	.26	0.81	1.24
**CRT-2**	-0.21	0.08	-0.09	-2.82	.005	0.90	1.11
***R***^***2***^ ***(Model 3)***		.41					
**Adjusted *R***^***2***^ ***(Model 3)***		.40					
***F (Model 3)***		22.39			< .001		

### Discussion

Data collected in Study 3 were consistent with both our hypotheses: people with higher scores on measures of positive schizotypy rated themselves as more likely to share examples of false political stories, as did people whose political information sharing was motivated by a desire to attack or manipulate others. Note however that these effects are small in magnitude, and arise from the correlational analyses. When other variables are included in the regression analyses (both pre-registered and exploratory) these effects become statistically non-significant. This suggests that the effects observed may arise from the variance these characteristics share with other more influential predictors, or that their effect is mediated by other variables included in the further analyses.

Pre-registered exploratory analyses indicated that a range of other variables had modest associations with either likelihood of sharing of false information in general, or likelihood of sharing stories known to be false. Multiple regression analyses indicated that the most important predictor of both of these was the general tendency to share political information. Right-leaning political ideology predicted overall but not deliberate sharing of false stories. This can be attributed to the fact that the stories themselves were all right-leaning; consistency between beliefs and the content of stories is known to be an important determinant of engagement [14, 44]. However, right-leaning ideology does not appear to predict deliberate deception. Among the motivational variables, sharing for reasons of raising awareness predicted both general and deliberate sharing of false stories, while sharing for entertainment purposes also predicted deliberate sharing of stories believed to be untrue. Notably, our measure of positive schizotypy did not emerge as a significant predictor in these regressions. Other variables appear to account for more variance in sharing stories. Tendency to share for the purpose of attacking or manipulating others was also non-significant, suggesting that its influence may be accounted for by variance shared with other motivational variables, or with tendency to share political material in general.

Our extended analyses including other predictors suggested that willingness to share false stories (but not stories explicitly known to be false) was also predicted by older age, male gender, and lower CRT-2 score. The finding for CRT-2 is consistent with the idea that people with lower tendencies towards cognitive reflection are more likely to share false material through inattention, not by design. However, CRT-2 score was also negatively associated with willingness to share true stories. Considering the differences and similarities between predictors of willingness to share both true and false stories is informative about whether the variables described here predict information-sharing behavior in general, or whether any are specific to sharing false information. Variables predicting both types of sharing were older age, general tendency to share political information, sharing for Awareness reasons, and lower cognitive reflection. However, male gender and right-wing political ideology predicted likely sharing of false, but not true, stories.

On a methodological note, also shown in Table 18 are point-biserial correlations between the self-report measures of having shared false information, and overall and deliberate sharing scores. While the effect sizes are small, all of these were positively correlated and statistically significant, providing evidence for a degree of shared variance in the different outcome measures used by these previous studies.

## Study 4

Studies 1 and 2 relied on self-reports of past sharing. This methodology has drawbacks: it is reliant on accurate testimony, and participants actually knowing that material they shared was inaccurate. Self-reports may also be subject to different influences than ‘live’ in-the-moment behavior. Study 3 improved on this, using a scenario-based measure where participants were asked to make decisions about whether they would share genuine examples of false information. However, this is still one step removed from actual behavior ‘in the wild’. Study 4 sought to address this, using more ecologically valid methodology to explore relationships between individual differences (e.g. in schizotypy), motivations for sharing political information, and participants’ actual sharing of false information. We hypothesized that social media users who have shared false political information on their personal Twitter accounts would have higher levels of Cognitive Perceptual schizotypy (Hypothesis 1), and that social media users who have shared false political information on their personal Twitter accounts would have higher scores on the ’Attack or manipulation of others’ motive for sharing political information (H2).

The study was originally pre-registered (https://osf.io/w4kne). However, in the early stages of data collection it became clear that we would not be able to achieve our target sample size due to low response rates in both stages. Trialling the protocol for stage 2 also indicated that data coding would be considerably more labor-intensive and time consuming than anticipated. Due to resource constraints, we therefore terminated data collection early and changed the analysis plan. All analyses should be considered as exploratory. Data, analysis code, and materials are available at https://osf.io/d84mu/. Ethical approval came from the University of Westminster College of Liberal Arts and Sciences Research Ethics Committee, approval code ETH2223-1847. Participant recruitment and data collection took place from May 11, 2023—June 18, 2023. Participants provided informed consent by selecting the appropriate option on the online questionnaire.

### Methods

#### Participants and recruitment

Participants were US-based Prolific users who share political material on social media. We recruited our sample in two stages to ensure that people who never shared political material were not included in our sample. First, we conducted a 1-minute pre-selection phase, *N* = 2999. Participants who reported (1) Non-zero levels of sharing political material on social media, and (2) having an active publicly visible English-language Twitter account personally operated for at least a year, which they used at least once a week, were invited in the debrief to take part in the second stage of the study.

The invitation stated that participants in the follow-up study would be asked for permission to look at material they had re-tweeted on Twitter. Participants could accept the invitation by sending our research team a private message on Twitter with their (auto-generated) anonymized individual participant code. Participants were informed that the purpose of this procedure was to enable us to link their public postings on Twitter with their responses to the follow-up questionnaire, which we would only do if they consented to take part in the follow-up study. A bonus of the $US equivalent of GBP £1 was paid to the 134 participants (including 9 who were invited using a parallel pilot procedure) who accepted our invitation and sent us a Twitter message.

These 134 were invited to take part in stage 2, and 120 did so in exchange for the $US equivalent of GBP £1.80-£7.20 (we gradually increased payment to encourage participation). Six responses were excluded based on our pre-registered criteria: three did not pass bot detection / fraud data quality checks embedded in Qualtrics, one reported a country of residence that was not the US and two had a zero-variance pattern of questionnaire completion that suggested inauthentic responses. We also excluded one user who tried to complete the study twice using two different Twitter accounts. Thus, our analytic sample consisted of 113 US residents. Demographic characteristics are shown in Table 1.

#### Procedure and materials

Data collection was carried out using Qualtrics. Participants completed the same background measures and social media measures as in Study 1, including self-reports of having shared false information accidentally and deliberately, with the addition of their 2020 US presidential election vote. Reasons for sharing political information online were measured, as in Study 2, with the 18-item questionnaire assessing 6 clusters of motives [33]. As in Studies 1 and 2, Cognitive Perceptual schizotypy was measured with the SPQ-BRU [35].

Participants then completed a range of other measures: psychopathy [42]; agreeableness [37]; cognitive reflection [36]; decision making style (rational and spontaneous) [23]; conscientiousness [37]; ‘fun’ humor style [43]; trait aggression [45]. These had been intended for use in exploratory analyses, but given the low achieved power are not considered further.

Real-life sharing of false information was assessed by coding each of the participants’ 100 most recent publicly available re-tweets and quote-retweets for whether they contained false political information. A retweet is where a user re-shares a post made by another person to their own follower network. A quote-retweet is where they do this, adding additional commentary of their own. Coding was done by reference to a custom database we prepared containing authoritative information compiled by third party-fact checkers. Full details of this process can be found in **S1 Appendix.** Our measure of real-life sharing of false information was calculated as the sum of posts we categorized as ‘false’, which people had shared without including debunking/opposing information.

### Results

Descriptive statistics for variables included in our analyses are shown in Table 24. Reliability for Attack or manipulation of others was very low in this sample, perhaps due to low levels of endorsement by participants. Of our 113 participants, the majority had not shared any false stories. Of the 17 (15%) who had shared false stories without trying to debunk them, 4 had shared one story, 6 had shared two, one each had shared 3, 4 or 5, and 2 each had shared 6 or 8 stories.

**Table 24:** Descriptive statistics for key variables, and Pearson’s *r* and Spearman’s *rho* correlations with number of false posts retweeted, Study 4 (*N* = 113).

	M	SD	Cron bach’s α	Range (potential)	Range (actual)	Skew	Kurtosis	Correlation with false tweets (*r*)	Correlation with false tweets (*rho*)
**Age**	38.89	13.14		18+	19–74	0.58	-0.53	.22*	.18
**Political** **ideology**	2.52	1.71		1–7	1–7	0.93	-0.17	.04	-.04
**Political** **information** **sharing**	2.41	1.09		1–5	1–5	0.52	-0.34	.33***	.31***
**Prosocial** **activism**	3.59	1.12	0.93	1–5	1–5	-0.79	0.00	.20*	.30**
**Attack or** **manipulation** **of others**	1.27	0.56	0.57	1–5	1–3.67	2.44	5.91	.10	.09
**Entertainment**	2.75	1.38	0.96	1–5	1–5	0.24	-1.24	.15	.07
**Awareness**	2.32	1.03	0.69	1–5	1–5	0.63	-0.15	.25**	.25**
**Political** **self-expression**	3.53	1.18	0.93	1–5	1–5	-0.66	-0.48	.14	.21*
**Fighting false** **information**	2.47	1.26	0.88	1–5	1–5	0.36	-1.04	.02	.10
**Cognitive** **Perceptual** **Schizotypy**	1.91	0.74	0.90	1–5	1–4.43	0.74	0.15	.13	.12
**Ideas of** **reference**	2.28	1.11	0.87	1–5	1–5	0.49	-0.90	.03	.07
**Suspicion**	2.10	1.02	0.81	1–5	1–5	0.83	0.01	.08	.11
**Magical** **thinking**	1.77	0.94	0.87	1–5	1–5	1.25	0.98	.22*	.21*
**Unusual** **perceptions**	1.62	0.80	0.79	1–5	1–4	1.12	0.25	.06	.00
**Number of** **false tweets** **shared**	0.50	1.49		0–100	0–8	3.64	13.56		
**Accidental** **sharing**								.23*	.32***
**Deliberate** **sharing**								-.06	-.09

Our original analysis plan was to compare individuals who had shared false stories with those who had not, in terms of their scores on measures of Cognitive Perceptual schizotypy and of sharing political material in order to attack or manipulate others. Independent samples *t-*tests indicated that Cognitive Perceptual schizotypy was not statistically significantly higher (*t*(111) = 1.33, *p* = .19, Hedges’ *g* = .35) for people who had shared false information (*M =* 2.13, *SD* = 0.85) than for those who had not (*M* = 1.82, *SD* = 0.72). Similarly, score on motivation to share political information to attack or manipulate others was not higher (*t*(111) = 1.12, *p* = 0.27, Hedges’ *g* = 0.29) for people who had shared false political stories (*M* = 1.41, *SD* = 0.74) than for those who had not (*M* = 1.25, *SD =* 0.52).

Reasoning that more information could be provided using the actual number of false stories retweeted by each participant, we also computed correlations between that variable, Cognitive Perceptual schizotypy, and motivations for sharing. We also included tendency to share political information online in this analysis, given its importance in Studies 1 and 3, political ideology, age, and self-reports of having shared false information in the past. Pearson’s correlations are shown in Table 24 (we also report Spearman’s *rho* given the non-normal distribution of the number of false tweets). On this occasion, we also scored the Cognitive Perceptual schizotypy measure for its constituent dimensions (Suspiciousness, Ideas of Reference, Magical Thinking, and Unusual Perceptions). This exploratory correlational analysis indicated that higher levels of sharing false information in real life were associated with higher scores on the Magical Thinking schizotypy subscale, sharing for reasons of Prosocial Activism and Awareness, tendency to share political information. Age correlated positively with false retweets in the Pearson’s, but not Spearman’s, correlations, while the reverse was true for sharing for reasons of Political Self-expression. Real world sharing of false information correlated positively with self-reports of accidental sharing, but not deliberate sharing (which only 15 people had reported in this sample).

### Discussion

While Study 4 was an underpowered exploratory analysis, a number of insights have emerged. Importantly, self-reported accidental sharing of false information correlated positively with the number of false tweets participants had actually shared without trying to debunk them. This provides some support for the validity of our measures in Studies 1 and 2.

Hypothesis 1, that those who shared false material would be higher in Cognitive Perceptual schizotypy, was not supported. However, there was a positive association between sharing and the Magical Thinking component of the schizotypy scale. This resonates with data from [46] who reported higher levels of belief in false news headlines among individuals more prone to delusional thinking, which they equate with the Magical Thinking measure used here. There is therefore a suggestion here that at least one aspect of positive schizotypy is relevant to real-life sharing of false information.

Two of the motivation variables–Prosocial Activism and raising Awareness–were related to real-life sharing of false material, potentially alongside Political Self-expression. Of these three, only Awareness—which had been associated with the amplification measure from Study 2 –had previously been found to be relevant. Neither the Entertainment nor Attack/manipulation motivations (H2), both of which were found to be relevant in Study 2, emerged as significant correlates of sharing here. The emerging picture is that motivations for sharing political information online do seem to be associated with sharing false information (both self-reported and actual), but that the relative importance of motivations other than raising awareness remains to be established.

Finally, tendency to share political information was most strongly associated with real-life sharing of false information. This reinforces the idea that it needs to be taken into account when considering determinants of sharing false material.

## General discussion

Across Studies 1–4, a number of patterns have emerged. In this general discussion, we focus primarily on variables found to be influential across multiple studies.

### Positive schizotypy

First, we have found evidence that positive schizotypy is related to sharing false information. In Studies 1 and 2 respectively, Cognitive Perceptual schizotypy was associated with self-reports of having shared political information later found to be false, and with amplification of false information over the past month. In extended analyses, it was also found to be associated with accidental sharing in Study 2 and with deliberate sharing in both Studies 1 and 2. In Study 3, we used two indices of positive schizotypy, and both were related to participants’ ratings of how likely they would be to share a selection of false stories (though this effect was weak and appeared to be attenuated by inclusion of other variables in regression analyses). In Study 4, one aspect of positive schizotypy–Magical Thinking–was related to actual sharing of false information on participants’ Twitter timelines.

One caveat here is that that while our studies using different methodological approaches have shown positive schizotypy does appear to be relevant to sharing false information, the effect sizes are small and vary across studies. A general observation here is that the effects of any variable on sharing misinformation are likely to be small: [1] identify 18 different categories of variables likely to influence spreading misinformation, of which personality is just one. Thus, any single personality variable is only likely to account for a very small portion of variance in misinformation sharing behavior.

To clarify how much of an effect positive schizotypy actually has, we calculated a meta-analytic effect size of SPQ-BRU Cognitive Perceptual schizotypy on self-reported sharing of information. Data about this from Prolific users was available from 5 different studies (Studies 1, 2 and 3 reported here, and two studies from [17]). For full information about this analysis see **S2 Appendix**. For the comparison of Cognitive Perceptual schizotypy scores between people who had not and who had shared false information by accident, the meta-analytic effect size was *d* = .46 (95% *CI* = .36-.55), with higher schizotypy levels in people who had done so in all studies. For sharing political stories which were known to be false at the time, the meta-analytic effect size was *d* = .73 (95% *CI* = .61-.84), again with higher levels among those who had shared in all studies. These effect sizes exceed the *d* = .41 threshold suggested for ‘practically meaningful’ effect sizes in social science research [47]. The effect size for deliberate sharing was influenced by one very high effect size. Winsorizing this reduced the overall meta-analytic effect size from .73 to .64, which is still substantive.

As noted previously, a potential explanation for the influence of positive schizotypy on accidental sharing is that positive schizotypy is associated with more intuitive than reflective/deliberate thought, and greater influence of heuristics and biases in decision making. However, the fact that positive schizotypy had an effect on self-reports in Studies 1 and 2, while scores on the cognitive reflection test (which should have a similar effect) did not in Study 1, suggests the reasons for its influence may be more complicated.

The fact that the effect size was weaker in Study 3, where there was greater scope for the operation of biases, suggests that there could be an effect operating in Studies 1 and 2 whereby people high on positive schizotypy are simply over-reporting that they have shared false information. This suggestion is reinforced by the evidence from the current research and the meta-analysis that positive schizotypy is also relevant to deliberate sharing of false information (with a larger effect size than for accidental sharing). This is less easy to explain than accidental sharing in terms of intuitive, rather than considered, processing. However, the fact that an aspect of schizotypy (Magical Thinking) correlated with objectively-measured real-life sharing of false information in Study 4 indicates that there is more going on than simple over-reporting. We have interpreted the findings of Study 4 as providing evidence that positive schizotypy in a broad sense is relevant to sharing false information, but it seems clear that further work is needed to investigate its precise effects, especially as only one aspect of schizotypy appeared to be influential in Study 4.

In Study 3, neither of our schizotypy measures correlated with accuracy of truth ratings, and MSS-B Positive Schizotypy did not predict likelihood of sharing true stories. In Studies 1 and 2, effects of schizotypy were still seen when general tendency to share political information was included in the regression analyses. Therefore, neither impaired veracity discernment nor greater overall likelihood of sharing information seem to be a complete alternative explanation for the results we report here.

### Motives

A second key finding is that motives are important determinants of sharing false information, and appear to play a larger role than individual difference variables. Across Studies 2–4, a number of different clusters of motives, drawn from a framework described by [33], were found to be relevant.

We had hypothesized that people motivated to share political information to attack or manipulate others would be more likely to share false information on purpose. Evidence consistent with this was found in Study 2, where people motivated by this reason were more likely to report themselves as having shared political stories they knew were false, and scored higher on the measure of false information amplification. While self-reports were not the primary focus of Study 3, Table 18 shows that this motive was associated with self-reports of having shared false information both accidentally and deliberately. This is consistent with [33] who report parallel findings using similar methodology. However, when included in regression analyses alongside other motivations and general tendency to share political information, the Attack motive did not emerge as a statistically significant predictor of the likelihood of sharing examples of false stories. Furthermore, it was not associated with actual sharing behavior in Study 4. This suggests that higher scores on the Attack motive are associated with self-reports of sharing false information, particularly deliberately, but not with in-the-moment decisions about what to share, or evidence of actual sharing behavior. It is possible that this variable may influence our views about whether it is acceptable to share false material, or honesty about doing so, but not the behavior itself.

The motivational variable that appeared most consistently across studies was sharing political information for ‘Awareness’ reasons. It is worth revisiting the ‘meaning’ of this motivational cluster. While labelled ‘Awareness’, it represents something more nuanced than just a desire to make people aware of some political information. It was described by [33]–who found it predicted self-reports of both deliberate and accidental sharing, though with a smaller set of predictors than the current work–as a prosocial motivation for sharing political information, reflecting a concern that ’voices be heard’ and with counteracting media biases. It also has somewhat of a conspiracist or paranoid overtone, with one of the items saying ‘people should know about secret plots planned against them’. This cluster of motives correlated *r*>.2 with self-reported accidental sharing of false information, and amplification, in Study 2. However, it did not emerge as a significant predictor in the Study 2 regression analyses, suggesting that it may share variance with one or more other predictors that attenuated or mediated its effect. The equivalent correlations with self-reported accidental and deliberate sharing were stronger in Study 3. It also emerged as a predictor of the overall likelihood of sharing false stories, and of sharing stories known to be false, in the Study 3 regression analyses. It is possible that the difference in the strength of correlations between Study 2 and 3 arises from differences in the two samples (mixed political views in Study 2, entirely right-wing in Study 3). The fact that Awareness had an effect in the Study 3 regressions (where decision to share individual stories was the outcome variable), but not Study 2 regressions (where the outcome variables were self-reports of past sharing) suggests that it may influence in-the-moment decisions about sharing more than self-reports. However the strongest piece of evidence for the importance of this motivational cluster comes from Study 4, where it correlated with real-life sharing of false stories. Does the fact that it also predicted likely sharing of true stories in Study 3 mean that it simply describes a tendency to share information, whether true or false? We would argue not, based on the facts that it correlated with self-reports of amplification of false stories (Study 2) and deliberate sharing of false stories (Study 3); that it predicted likely sharing of stories *known* to be false (Study 3); and that it predicted likelihood of sharing false stories (whether known to be false or not) even when general tendency to share political information was controlled for.

While Attack/manipulation and Awareness appear to be the most important predictors of self-reported or actual sharing, they are not among those rated as most important by individuals in Study 2 who completed the motivation scales relative to false stories they had actually shared. These suggested Prosocial Activism and Political Self-expression were considered most important for accidental sharing, and Entertainment and Fighting False Information were most important for deliberately sharing a false story. While it is possible that respondents were attempting to present themselves in a positive light in Study 2, there is a discrepancy here. It is also important to note that the motivational variables are expected to intercorrelate [33]. The variance they share is clearly seen in their similar relationships with the indices of sharing false information in Table 18 for example.

### Other variables

We also note strong evidence that general tendency to share political information is associated with higher levels of sharing false stories, both deliberately and accidentally. This is unsurprising: the more one tends to share false political stories, the greater one’s level of political sharing will be. Some of the other variables under consideration are likely to correlate or interact with level of political sharing: people who share political stories must after all be motivated to do so. All of the motivational variables considered will predict it to some extent (for example in Study 3, correlations between each of the motivation variables and general tendency to share political material ranged from *r* = .31 to *r* = .64). It is likely that this should be considered as a possible mediating variable in any study looking at person-level predictors of engagement with false information.

Findings for cognitive reflection, operationalized with the CRT-2, were mixed in terms of their consistency with our hypotheses. In Study 1, CRT-2 scores were not associated with self-reports of sharing false information. However, as previously noted the methodology of this study may not have provided scope for the effect of cognitive reflection to operate: people were self-reporting past behavior, rather than making in-the-moment judgements about sharing that would be influenced by intuitive vs. considered decision making. Study 3, where people were asked to decide whether they would want to share specific stories, did offer the opportunity for the effect to operate. Our extended analyses indicated that people with lower levels of cognitive reflection said they were more likely to share false stories overall, but not stories they actually knew were false. This is consistent with broad theoretical expectations. However, CRT-2 scores did not correlate with accuracy of identifying stories as true or false, and a stronger link was actually observed with sharing true stories.

Finally, we did not find relationships between our measures of sharing false stories and several constructs that have previously been noted in the literature: notably, agreeableness, conscientiousness and need for chaos. This was despite the fact that Studies 1–3 were well-powered. If one adopts a benchmark of *r =* .2 for a ‘practically meaningful’ effect [47], the sample sizes in each of those studies conferred over 99% power to detect such an effect with alpha = .05. It is possible that previous findings obtained with these variables are actually due to variance they share with other constructs included in our analysis. For example, in Study 1, Cognitive Perceptual schizotypy correlated *r* = .35 (*n* = 614, *p* < .001) with Need for Chaos and *r* = -.23 (*n =* 614, p < .001) with Conscientiousness. In Study 3 it correlated *r = -*.35 (*n* = 627, *p* < .001) with Conscientiousness and *r =* .45 (*n* = 627, *p* < .001) with Agreeableness. This suggests that studies which consider multiple individual differences simultaneously, and consider how they may operate together, would provide a more accurate picture than those focusing on specific individual variables in isolation. Based on the current findings, such studies should include a measure of positive schizotypy.

### Limitations

There are a few limitations in this research that should be acknowledged. First is the fact that we relied extensively on self-report data rather than behavioral observations. The extent to which person-level variables influence reporting of past behavior may be different from the extent to which they influence behavior as it happens. They also rely on accurate memory of past behaviors, and in this case awareness that a story was actually false. However, we found convergent evidence for the different variables we used (see Study 3) and also between self-reports of having accidentally shared false material and actual real-world sharing of false stories in Study 4. Alongside evidence that self-reported willingness to share material correlates with actual real-world sharing [48], this allows a degree of confidence in the findings.

Second, the sample size obtained in Study 4 was too low to permit our planned analyses and enabled only a limited exploratory analysis to be performed. However, we note that Study 4 also presented a more granular analysis of real-world sharing than is often the case in this vein of research. Much published work that seeks to link individual characteristics to individual social media behavior has relied on analyses of whether known ‘fake news domains’ are referenced in individuals’ social media feeds [e.g. 16]. The work reported here coded for the presence of false stories at the level of individual postings by participants.

Third, there are some issues with the representativeness of the samples we used. While it is common to use participant panels such as Prolific in this type of research, we must remain alert to the characteristics of our participants. The samples in the studies reported here varied in their composition. For example, Study 3’s sample was comprised completely of Republican voters while Study 4’s sample comprised largely people who voted Democrat in 2020 (for comparison purposes with Study 3, a version of the Study 1 and 2 analyses restricted to right-wing participants can be seen in **S3 Appendix)**. If there are effects that are particular to people with a particular political orientation, they may be missed if there are few such people in the sample, or exaggerated if there are many. However, the fact that convergent findings were obtained across these samples suggests there is a degree of generalizability of findings.

Fourth, one might also ask whether the samples used here are representative of the general population in terms of the characteristics we have found to be important. For example, could our findings for positive schizotypy arise because our participants are particularly high on that trait, and the construct would not influence sharing false information in the broader population? In fact, the samples reported here are not unusually high in terms of schizotypy. The mean scores for Studies 1–4 are comparable to, or lower than, norms for Cognitive Perceptual schizotypy for a large sample of psychology undergraduates [35].

A final point relates to the way we have operationalized the terms ‘misinformation’ and ‘disinformation’, in line with formal definitions that specify them as material that is actually untrue. This is reflected in the self-report questions we used. However, there are other related categories of problematic material: for example, information that is technically true but is presented out of context in order to convey a misleading impression (sometimes called ’malinformation’; see e.g. [49] for a detailed discussion of these and related issues). The extent to which the current findings apply to such other types of misleading material is open to question.

### Practical implications

This work has implications for future work on misinformation. We have shown that motivations are an important influence on sharing false information, with both prosocial (e.g. raising awareness) and antisocial (attacking or manipulating others) motivations coming into play. Motivational variables may matter more than individual differences (e.g. in personality). This may well have implications for the design of anti-misinformation interventions. For example, content-based strategies such as fact-checking or debunking might be particularly relevant to individuals whose sharing is motivated by Fighting False Information. However, it is more difficult to see what strategies might work for individuals with antisocial motivations, other than platform-level interventions such as banning.

Future work needs to take general sharing of political information into account, given its emergence as an important predictor of sharing false stories both accidentally and deliberately. As well as being influenced by motivation, it may also mediate the role of individual difference variables (cf. [17] who found it mediated the effect of psychopathy on self-reports of sharing false stories).

Finally, the role of positive schizotypy needs to be considered further. There is currently a tangled picture of how different personality variables influence engagement with misinformation. In the current data, we found evidence that positive schizotypy, but not previously investigated variables (need for chaos, agreeableness, conscientiousness) or other trait constructs (aggression, decision making style, humor style), was relevant to sharing false information. However, as noted above there are also questions to be answered about how any such ‘schizotypy effect’ actually operates, and under what kind of circumstances it is likely to be observed.

## Conclusion

In summary, our four studies provide evidence that positive schizotypy is associated with measures of sharing false political information. It emerges as more important than any of the personality, cognitive style, or other individual differences we considered. They also provide evidence for the importance of motivation. While a range of motivations for sharing political information online were associated with sharing false information, two appeared particularly important: a desire to share political stories to attack or manipulate others, and to share political stories in order to raise awareness. While individuals reported different motivations for sharing specific false stories, these two factors appeared to influence both deliberate and accidental sharing of false stories. However, it is possible that the Attack motive is associated more with views about whether it is acceptable to share false information, or increased reporting of doing so, rather than the behavior itself. Understanding the role of motivation in more detail, as well as the effects of positive schizotypy, are likely to be productive themes for future misinformation research.

## Supporting information

S1 Appendix Coding of participant retweets. (DOCX)

S2 Appendix Meta-analyses. (DOCX)

S3 Appendix Study 1 and 2 analyses for right-wing participants. (DOCX)
