# Supplementary material for: Individual differences in sharing false political information on social media: Deliberate and accidental sharing, motivations and positive schizotypy
Source: PLoS One. 2024 Jun 26;19(6):e0304855. doi: 10.1371/journal.pone.0304855 (PMC11206957; doi:10.1371/journal.pone.0304855)
Supplement: S1 Appendix — (DOCX) [file pone.0304855.s001.docx]

**Supporting information**

**S1 Appendix 1: Coding of participant retweets**

Real-life sharing of false information was assessed by coding each of the participants’ 100 most recent publicly available re-tweets (RTs) and quote-retweets (QRTs) for whether they contained false political information. An RT is where a user re-shares a post made by another person to their own follower network. A QRT is where they do this, adding additional commentary of their own. We only coded RTs and QRTs because we focused on sharing of existing false information, rather than generation of novel disinformation posts. Coding was done by reference to a custom database we prepared containing authoritative information compiled by third party-fact checkers.

**Preparation of database**

Our database included publicly available information from seven fact-checkers: factcheck.afp.com, checkyourfact.com, leadstories.com, snopes.com, factcheck.org, politifact.com, and logically.ai. We selected fact-checking websites that 1. focus on assessing political news either in the US or internationally; 2. were verified signatories of the International Fact-Checking Network code of principles at time of data scraping (March 2023) or were verified signatories during at least part of 2021-2; 3. have coverage of contested stories during 2021-2022; 4. use informative titles for stories that enable the identification of searchable keywords; and 5. present a list of stories in chronological order that can be scraped.

Next, we used Data Miner (a Chrome extension) to scrape information concerning contested stories (i.e., title, date of story) from each database for the calendar years of 2021-2022. We then aggregated data from the seven websites into a single Excel-format database that provided broad coverage of false political news stories (19,222 items in total).

**Coding of retweets: Overview**

We scraped publicly-shared information from each participant’s Twitter feed using [www.vicinitas.io](https://www.vicinitas.io/free-tools/download-user-tweets) – a Twitter analytics tool that provided this function at the time of the study (this function is no longer available). We used the downloaded feeds to prepare, for each participant, a list of posts that were: 1. Retweets (RTs) /Quote-retweets (QRTs), and 2. Dated between 2021-2022. We then applied our coding protocol to each of these lists.

Drawing on [1], two independent coders identified keywords for each shared story and manually searched our Excel-format database for fact-checking information concerning the same story to code the stories as *False*, or *False with opposing information* (where the story was shared by the participant criticised or debunked it). Disagreements were settled by consensus with a third reviewer. Real-life sharing of false information for each participant was calculated as the number of posts rated as *False*.

**Detailed coding protocol and procedural information**

1. For each user, we extracted RTs/QRTs made within the calendar years 2021-22, and examined the latest 100 RTs and QRTs within this period (i.e., backwards from 31.12.2022).

2. Drawing on [2] we defined "false" stories as either *de-contextualized or partially false information* OR *fabricated or completely false information*.

3. The coding procedure for each participant involved two independent coders (from a pool of three) who reviewed pre-prepared Excel files containing the participant’s latest 100 RTs and QRTs.

4. For each RT/QRT, each coder rated whether it contained (or did not contain) political information. We adopted an intentionally broad set of criteria intended to identify whether a tweet contained 'political' material. We considered political material to reference one or more of the following:

- 1. Individuals associated with politics, either directly (e.g., Joe Biden) or by association (e.g., Hunter Biden).
  2. Political parties (e.g., Republicans, Democrats)
  3. Formal or non-formal organisations that have links with political platforms or movements (e.g. NRA, Proud Boys).
  4. Political processes (e.g., elections) or organisations involved in their administration (e.g., Dominion voting machines)
  5. Government entities (legislature, executive, judiciary) at either national or local level.
  6. Geopolitical interactions (e.g., wars, international political summits or organisations)
  7. Politically sensitive or politicised issues (e.g. abortion, gun control, vaccination, masks, civil rights and equality issues, taxation, budgets, immigration, gender issues, climate change, public health, education)
  8. Political ideologies (e.g., conservatism, libertarianism)
  9. Politicised terms or slogans (e.g., MAGA, RINO, 'Woke', black lives matter, critical race theory)
  10. Symbols or images known to have political connotations (e.g., Pepe the Frog, Confederate flags)

5. Disagreements as to whether a tweet contained political information were settled by discussion between the two coders until consensus was reached.

6. If a tweet contained a video/document/image/link, each coder watched (the first five minutes of any video)/read it closely to identify the main claim being made (however, see 6a for exceptions).

6a. Safeguarding measures: If RT/QRT was flagged by a warning (e.g., age-sensitive material, difficult images) OR it could be inferred from information in the post that the content was potentially disturbing (e.g., violent theme), coders did not engage with the content (this included text, images, and video).

7. If RT/QRT did not contain political information, coder proceeded to next item.

8. If RT/QRT contained political information:

- 1. Coder marked post as containing political information.
  2. Coder extracted and recorded 1 keyword (or phrase e.g., minimum wage) that was essential to the headline.
  3. Where coders identified multiple potential false claims in a tweet, a keyword was identified and checked for each claim it contained.
  4. When the story was not judged as including a clear essential keyword (e.g., Trump), coders tested 1-3 keywords against the database.
  5. Coders were instructed to pay attention to context/theme of story (e.g., Covid, elections).
  6. Coders searched for keyword(s) in complete database OR in subsets of stories containing common, selected keywords (e.g., ‘Trump’, ‘Musk’).
  7. Coders considered matches against original heading, keyword, and context/theme.
  8. If a potential match, coder examined the fact-check (via searching for the title online and/or adding the name of fact-checker).
  9. Fact check was considered relevant if it examined the same claim as made in the RT/QRT or a closely related claim (based on content/theme).
  10. Any information found in fact-check was treated as ‘true’, and used to either support or invalidate the claim being made in the tweet.
  11. Based on the information in the fact-check, coders marked each RT/QRT as FALSE or NOT FALSE or FALSE WITH OPPOSING INFORMATION (e.g., debunking). Any disagreements between coders were settled by discussion with a third independent coder until consensus was reached.
  12. If post was FALSE (or False with opposing information), coder recorded a link to the relevant fact-check.

7. We examined only RTs/QRTs in English.

8. Our procedure did not capture information that is limited by technical constraints (e.g., RT/QRTs that involved media that was unavailable/ had been removed). It was also restricted to identifying information that appear in fact-checking websites.

**References**

1. Charquero-Ballester M, Walter JG, Nissen IA, Bechmann A. Different types of COVID-19 misinformation have different emotional valence on Twitter. Big Data & Society. 2021;8: 205395172110412.
2. Hameleers M, Humprecht E, Möller J, Lühring J. Degrees of deception: the effects of different types of COVID-19 misinformation and the effectiveness of corrective information in crisis times. Information, Communication & Society. 2023;26: 1699-1715.
