# Supplementary material for: Individual differences in sharing false political information on social media: Deliberate and accidental sharing, motivations and positive schizotypy
Source: PLoS One. 2024 Jun 26;19(6):e0304855. doi: 10.1371/journal.pone.0304855 (PMC11206957; doi:10.1371/journal.pone.0304855)
Supplement: S2 Appendix — (DOCX) [file pone.0304855.s002.docx]

**Supporting information**

**S2 Appendix 2: Meta-analyses**

We conducted two separate mini meta-analyses on the effect of cognitive perceptual schizotypy on 1. accidental historical sharing of false political stories (Table A2.1) based on answers to the question “Have you ever shared a political news story online that you later found out was made up? (yes/no)”, and 2. deliberate historical sharing of false political stories (Table A2.2) based on answers to the question “And have you ever shared a political news story online that you thought AT THE TIME was made up? (yes, no)”.

We included data from five studies that used identical measures for the constructs under study: three from this paper, and two from [1]. A positive effect size indicates higher levels of cognitive perceptual schizotypy among participants who reported historical sharing (accidental, deliberate) in comparison to those who reported no historical sharing. We used Hedges’ *g* effect size indicator due to the unbalanced size of comparison groups. Because the study population was identical across five of our studies (i.e., US-based Prolific users without additional selection stages), we a-priori selected a fixed-effects model; this model enables making inferences only to this particular population [2]. Study 4 had a sample that went through an additional self-selection process and was therefore excluded from the meta-analysis to enhance comparability of the included studies. Effect sizes were calculated using SPSS 28.0.1.1, based on *M*, *SD*s, and *n* per group, drawn from publicly shared summary data. Where Levene’s test for equality of variances was significant at *p*<.05, the *t* value under equal variances not assumed is reported. The meta-analysis was conducted on the same software using syntax provided by [3].

**Table A2.1. Mini meta-analysis of cognitive perceptual schizotypy scores for people who reported they had or had not accidentally shared false political information.**

|  | **Had not accidentally shared** | **Had accidentally shared** |  |  |
| --- | --- | --- | --- | --- |
|  | *M (SD) / n* | *M (SD) / n* | *t* | Hedge’s *g* (95% CI) |
| ***Buchanan & Kempley, 2021*** |  |  |  |  |
| **Study 1** | 27.69 (9.32) / 404 | 33.03 (12.53) / 103 | 4.05*** | .53 (.31 – .75) |
| **Study 2** | 27.38 (10.31) / 424 | 35.25 (14.64) / 103 | 5.15*** | .70 (.48 – .92) |
| ***Buchanan et al., current paper*** |  |  |  |  |
| **Study 1** | 2.15 (.63) / 513 | 2.42 (.74) / 101 | 3.46*** | .42 (.20 – .63) |
| **Study 2** | 2.16 (.66) / 435 | 2.42 (.71) / 127 | 3.89*** | .39 (.19 – .59) |
| **Study 3** | 1.74 (.67) / 495 | 1.95 (.75) / 132 | .30** | .31 (.12 – .50) |
| **Weighted mean effect size** | *d* = .46, 95% CI = (.36, .55) | | | |

***p* < .01

****p* < .001

**Table A2.2. Mini meta-analysis of cognitive perceptual schizotypy scores for people who reported they had or had not deliberately shared false political information.**

|  | **Had not deliberately shared** | **Had deliberately shared** |  |  |
| --- | --- | --- | --- | --- |
|  | *M (SD) / n* | *M (SD) / n* | *t* | Hedge’s *g* (95% CI) |
| ***Buchanan & Kempley, 2021*** |  |  |  |  |
| **Study 1** | 27.84 (9.56) / 444 | 35.38 (12.52) / 63 | 4.60*** | .76 (.49 – 1.02) |
| **Study 2** | 27.33 (10.33) / 463 | 40.39 (14.42) / 64 | 7.00*** | 1.20 (.93 – 1.47) |
| ***Buchanan et al., current paper*** |  |  |  |  |
| **Study 1** | 2.16 (.64) / 552 | 2.51 (.69) / 62 | 3.95*** | .53 (.26 – .79) |
| **Study 2** | 2.18 (.65) / 495 | 2.51 (.82) / 67 | 3.21** | .50 (.24 – .76) |
| **Study 3** | 1.74 (.67) / 570 | 2.20 (.82) / 57 | 4.15** | .68 (.41 – .96) |
| **Weighted mean effect size** | *d* = .73, 95% CI = (.61, .84) | | | |

***p* < .01

****p* < .001

The estimates of effect sizes in US-based Prolific users were statistically significant for both types of historical sharing. For accidental historical sharing, a chi-square test of homogeneity was non-significant, *p* = .10, suggesting that the effect sizes in included studies are roughly equivalent and so the effect sizes are likely homogeneous among US-based Prolific users. The weighted mean effect size for the relationship between cognitive perceptual schizotypy and accidental historical sharing was .46, representing a medium effect [4]. For deliberate historical sharing, the homogeneity test was significant, *χ*^2^(4) = 16.87, *p* < .01, suggesting that there is variation in effect sizes in included studies. The weighted mean effect size for the relationship between cognitive perceptual schizotypy and deliberate historical sharing was .73, representing a medium-large effect [4]. Winsorizing (i.e., replacing with the next highest effect size) [5] one very high effect size (*g* = 1.20) reduced the overall mean from .73 to .64 but did not affect its statistical significance.

**References**

1. Buchanan T, Kempley J. Individual differences in sharing false political information on social media: Direct and indirect effects of cognitive-perceptual schizotypy and psychopathy. Personality and Individual Differences. 2021;182: 111071.

2. Borenstein M, Hedges LV, Higgins JP, Rothstein HR. A basic introduction to fixed-effect and random-effects models for meta-analysis. Research Synthesis Methods. 2010;1: 97-111.

3. Field AP, Gillett R. How to do a meta-analysis. British Journal of Mathematical and Statistical Psychology. 2010;63: 665-694.

4. Cohen J. A power primer. Psychological Bulletin. 1992;112: 155 - 159.

5. Lipsey MW, Wilson DB. Practical Meta-Analysis. Thousand Oaks: SAGE Publications, Inc.; 2001.
