# Supplementary material for: Individual differences in sharing false political information on social media: Deliberate and accidental sharing, motivations and positive schizotypy
Source: PLoS One. 2024 Jun 26;19(6):e0304855. doi: 10.1371/journal.pone.0304855 (PMC11206957; doi:10.1371/journal.pone.0304855)
Supplement: S3 Appendix — (DOCX) [file pone.0304855.s003.docx]

**Supporting information**

**S3 Appendix 3: Study 1 and 2 analyses for right-wing participants**

Study 1 and 2 included participants with a mix of political orientations, while Study 3 contained exclusively Republican voters. To enable comparison between the studies, we repeated the extended regression analyses from Studies 1 and 2 with right-wing participants only. This was operationalised by selecting individuals who scored above the midpoint of the item *“Some people describe political affiliation on a left to right spectrum. Please indicate where you believe your political ideology lies on this spectrum.”,* which participants responded to on a seven-point scale with the response options “*1 (left), 2, 3, 4 (center), 5, 6, 7 (right)*”. Note that where there are differences between the findings shown here, and the full samples, this could be due either to differences in the sample characteristics or the reduced statistical power available due to the lowered sample sizes.

**Table A3.1. Binary logistic regression: Predictors of whether participants had previously shared political stories they subsequently discovered were false, Study 1 (all predictors, right wing participants only, *N*=234).**

|  | ***B*** | ***S.E.*** | ***Wald*** | ***df*** | ***p*** | ***Exp(B)*** | **95% C.I. for *EXP(B)*** |
| --- | --- | --- | --- | --- | --- | --- | --- |
| **Step 1** |  |  |  |  |  |  |  |
| **Age** | 0.02 | 0.01 | 3.62 | 1 | .06 | 1.02 | [1.00, 1.05] |
| **Political ideology (L-R)** | 0.29 | 0.24 | 1.51 | 1 | .22 | 1.34 | [0.84, 2.13] |
| **Gender (M=1, F=2)** | 0.09 | 0.41 | 0.04 | 1 | .83 | 1.09 | [0.49, 2.43] |
| **Constant** | -4.34 | 1.58 | 7.52 | 1 | .01 | 0.01 |  |
| **Step 2** |  |  |  |  |  |  |  |
| **Age** | 0.03 | 0.01 | 4.19 | 1 | .04 | 1.03 | [1.00, 1.05] |
| **Political ideology (L-R)** | 0.29 | 0.24 | 1.36 | 1 | .24 | 1.33 | [0.82, 2.15] |
| **Gender (M=1, F=2)** | 0.10 | 0.42 | 0.05 | 1 | .82 | 1.10 | [0.48, 2.52] |
| **Political information sharing** | 0.56 | 0.17 | 10.68 | 1 | .001 | 1.74 | [1.25, 2.43] |
| **Constant** | -5.46 | 1.69 | 10.42 | 1 | .001 | 0.00 |  |
| **Step 3** |  |  |  |  |  |  |  |
| **Age** | 0.03 | 0.01 | 5.90 | 1 | .02 | 1.04 | [1.01, 1.06] |
| **Political ideology (L-R)** | 0.26 | 0.25 | 1.07 | 1 | .30 | 1.30 | [0.79, 2.13] |
| **Gender (M=1, F=2)** | 0.14 | 0.47 | 0.08 | 1 | .77 | 1.15 | [0.45, 2.90] |
| **Political information sharing** | 0.54 | 0.18 | 9.46 | 1 | .00 | 1.72 | [1.22, 2.44] |
| **Need for Chaos** | -0.17 | 0.42 | 0.16 | 1 | .69 | 0.85 | [0.37, 1.93] |
| **Spontaneous Decision-Making** | 0.44 | 0.32 | 1.89 | 1 | .17 | 1.55 | [0.83, 2.91] |
| **Rational Decision-Making** | 0.00 | 0.43 | 0.00 | 1 | 1.00 | 1.00 | [0.43, 2.33] |
| **CRT-2** | 0.21 | 0.20 | 1.15 | 1 | .28 | 1.24 | [0.84, 1.83] |
| **Conscientiousness** | 0.61 | 0.35 | 3.05 | 1 | .08 | 1.85 | [0.93, 3.68] |
| **Cognitive Perceptual schizotypy** | 0.59 | 0.35 | 2.85 | 1 | .09 | 1.81 | [0.91, 3.61] |
| **Constant** | -10.82 | 3.46 | 9.79 | 1 | .002 | 0.00 |  |

**Table A3.2. Binary logistic regression: Predictors of whether participants had previously shared political stories they knew at the time were false, Study 1 (all predictors, right wing participants only, *N*=234).**

|  | ***B*** | ***S.E.*** | ***Wald*** | ***df*** | ***p*** | ***Exp(B)*** | ***95% C.I. for EXP(B)*** |
| --- | --- | --- | --- | --- | --- | --- | --- |
| **Step 1** |  |  |  |  |  |  |  |
| **Age** | -0.02 | 0.02 | 1.33 | 1 | .25 | 0.98 | [0.95, 1.01] |
| **Political ideology (L-R)** | -0.45 | 0.30 | 2.22 | 1 | .14 | 0.64 | [0.36, 1.15] |
| **Gender (M=1, F=2)** | 0.22 | 0.47 | 0.21 | 1 | .64 | 1.24 | [0.50, 3.11] |
| **Constant** | 0.74 | 1.90 | 0.15 | 1 | .70 | 2.09 |  |
| **Step 2** |  |  |  |  |  |  |  |
| **Age** | -0.02 | 0.02 | 0.88 | 1 | .35 | 0.98 | [0.95, 1.02] |
| **Political ideology (L-R)** | -0.53 | 0.32 | 2.68 | 1 | .10 | 0.59 | [0.31, 1.11] |
| **Gender (M=1, F=2)** | 0.18 | 0.50 | 0.13 | 1 | .72 | 1.20 | [0.45, 3.18] |
| **Political information sharing** | 0.88 | 0.19 | 21.04 | 1 | <.001 | 2.42 | [1.66, 3.53] |
| **Constant** | -0.68 | 2.06 | 0.11 | 1 | .74 | 0.51 |  |
| **Step 3** |  |  |  |  |  |  |  |
| **Age** | -0.01 | 0.02 | 0.41 | 1 | .52 | 0.99 | [0.95, 1.03] |
| **Political ideology (L-R)** | -0.52 | 0.33 | 2.47 | 1 | .12 | 0.60 | [0.31, 1.14] |
| **Gender (M=1, F=2)** | 0.44 | 0.54 | 0.65 | 1 | .42 | 1.55 | [0.53, 4.49] |
| **Political information sharing** | 0.89 | 0.20 | 19.64 | 1 | <.001 | 2.42 | [1.64, 3.59] |
| **Need for Chaos** | 0.40 | 0.32 | 1.61 | 1 | .20 | 1.50 | [0.80, 2.79] |
| **Spontaneous Decision-Making** | 0.30 | 0.39 | 0.62 | 1 | .43 | 1.35 | [0.64, 2.88] |
| **Rational Decision-Making** | 0.12 | 0.50 | 0.06 | 1 | .81 | 1.13 | [0.42, 3.03] |
| **CRT-2** | 0.12 | 0.24 | 0.23 | 1 | .63 | 1.12 | [0.70, 1.81] |
| **Conscientiousness** | 0.21 | 0.40 | 0.27 | 1 | .60 | 1.23 | [0.56, 2.69] |
| **Cognitive Perceptual schizotypy** | 0.26 | 0.40 | 0.42 | 1 | .52 | 1.30 | [0.59, 2.84] |
| **Constant** | -4.84 | 4.00 | 1.46 | 1 | .23 | 0.01 |  |

**Table A3.3. Ordinal Regression: Predictors of Amplification of False Stories, Study 1 (all predictors, right wing participants only, *N*=234).**

|  | ***Estimate*** | ***Std. Error*** | ***Wald*** | ***df*** | ***Sig.*** | ***95% C.I. for Estimate*** |
| --- | --- | --- | --- | --- | --- | --- |
| **Age** | -0.03 | 0.02 | 2.52 | 1 | .11 | [-0.06, 0.01] |
| **Political ideology (L-R)** | 0.00 | 0.26 | 0.00 | 1 | .99 | [-0.50, 0.50] |
| **Gender (M=1, F=2)** | -0.36 | 0.43 | 0.70 | 1 | .40 | [-1.21, 0.49] |
| **Political information sharing** | 0.85 | 0.17 | 23.93 | 1 | <.001 | [0.51, 1.19] |
| **Need for Chaos** | 0.10 | 0.31 | 0.11 | 1 | .75 | [-0.51, 0.72] |
| **Spontaneous Decision-Making** | 0.14 | 0.32 | 0.20 | 1 | .66 | [-0.48, 0.76] |
| **Rational Decision-Making** | -0.53 | 0.42 | 1.59 | 1 | .21 | [-1.36, 0.29] |
| **CRT-2** | 0.11 | 0.20 | 0.32 | 1 | .58 | [-0.28, 0.50] |
| **Conscientiousness** | 0.04 | 0.32 | 0.01 | 1 | .91 | [-0.60, 0.67] |
| **Cognitive Perceptual schizotypy** | 0.17 | 0.34 | 0.24 | 1 | .62 | [-0.50, 0.84] |

**Table A3.4. Binary logistic regression: Predictors of whether participants had previously shared political stories they subsequently discovered were false, Study 2 (all predictors, right wing participants only, *N=*250).**

|  | ***B*** | ***S.E.*** | ***Wald*** | ***df*** | ***p*** | ***Exp(B)*** | **95% C.I. for *Exp(B)*** |
| --- | --- | --- | --- | --- | --- | --- | --- |
| **Step 1** | 0.01 | 0.01 | 0.36 | 1 | .55 | 1.01 | [0.99, 1.03] |
| **Age** | -0.52 | 0.30 | 2.98 | 1 | .08 | 0.60 | [0.33, 1.07] |
| **Gender (M=1, F=2)** | -0.20 | 0.20 | 1.00 | 1 | .32 | 0.82 | [0.55, 1.21] |
| **Political ideology** | 0.17 | 1.14 | 0.02 | 1 | .88 | 1.19 |  |
| **Constant** | 0.01 | 0.01 | 0.36 | 1 | .55 | 1.01 | [0.99, 1.03] |
| **Step 2** |  |  |  |  |  |  |  |
| **Age** | 0.00 | 0.01 | 0.07 | 1 | .79 | 1.00 | [0.98, 1.02] |
| **Gender (M=1, F=2)** | -0.41 | 0.31 | 1.73 | 1 | .19 | 0.66 | [0.36, 1.22] |
| **Political ideology** | -0.26 | 0.21 | 1.51 | 1 | .22 | 0.77 | [0.51, 1.17] |
| **Political information sharing** | 0.54 | 0.14 | 15.23 | 1 | <.001 | 1.72 | [1.31, 2.26] |
| **Constant** | -0.57 | 1.21 | 0.22 | 1 | .64 | 0.57 |  |
| **Step 3** |  |  |  |  |  |  |  |
| **Age** | 0.01 | 0.01 | 0.34 | 1 | .56 | 1.01 | [0.98, 1.03] |
| **Gender (M=1, F=2)** | -0.36 | 0.34 | 1.11 | 1 | .29 | 0.70 | [0.36, 1.36] |
| **Political ideology** | -0.45 | 0.24 | 3.50 | 1 | .06 | 0.64 | [0.40, 1.02] |
| **Political information sharing** | 0.04 | 0.19 | 0.05 | 1 | .83 | 1.04 | [0.72, 1.50] |
| **Prosocial activism** | 0.22 | 0.18 | 1.48 | 1 | .22 | 1.25 | [0.87, 1.78] |
| **Attack or manipulation of others** | 0.01 | 0.24 | 0.00 | 1 | .98 | 1.01 | [0.63, 1.60] |
| **Entertainment** | 0.19 | 0.15 | 1.67 | 1 | .20 | 1.21 | [0.91, 1.60] |
| **Awareness** | 0.13 | 0.23 | 0.31 | 1 | .58 | 1.14 | [0.72, 1.79] |
| **Political self-expression** | 0.23 | 0.19 | 1.46 | 1 | .23 | 1.26 | [0.87, 1.84] |
| **Fighting false information** | 0.33 | 0.18 | 3.47 | 1 | .06 | 1.39 | [0.98, 1.96] |
| **Cognitive Perceptual schizotypy** | 0.48 | 0.25 | 3.56 | 1 | .06 | 1.61 | [0.98, 2.64] |
| **Constant** | -2.82 | 1.53 | 3.39 | 1 | .07 | 0.06 |  |

**Table A3.5. Binary logistic regression: Predictors of whether participants had previously shared political stories they thought at the time were false, Study 2 (all predictors, right wing participants only, *N=*250).**

|  | ***B*** | ***S.E.*** | ***Wald*** | ***df*** | ***p*** | ***Exp(B)*** | **95% C.I. for *Exp(B)*** |
| --- | --- | --- | --- | --- | --- | --- | --- |
| **Step 1** | -0.02 | 0.01 | 1.10 | 1 | .29 | 0.99 | [0.96, 1.01] |
| **Age** | -1.81 | 0.51 | 12.69 | 1 | <.001 | 0.16 | [0.06, 0.44] |
| **Gender (M=1, F=2)** | -0.04 | 0.26 | 0.02 | 1 | .89 | 0.96 | [0.58, 1.60] |
| **Political ideology** | -0.45 | 1.51 | 0.09 | 1 | .77 | 0.64 |  |
| **Constant** | -0.02 | 0.01 | 1.10 | 1 | .29 | 0.99 | [0.96, 1.01] |
| **Step 2** |  |  |  |  |  |  |  |
| **Age** | -0.02 | 0.01 | 1.50 | 1 | .22 | 0.98 | [0.96, 1.01] |
| **Gender (M=1, F=2)** | -1.73 | 0.51 | 11.51 | 1 | <.001 | 0.18 | [0.07, 0.48] |
| **Political ideology** | -0.10 | 0.27 | 0.13 | 1 | .72 | 0.91 | [0.54, 1.54] |
| **Political information sharing** | 0.43 | 0.18 | 5.95 | 1 | .02 | 1.54 | [1.09, 2.18] |
| **Constant** | -0.99 | 1.57 | 0.40 | 1 | .53 | 0.37 |  |
| **Step 3** |  |  |  |  |  |  |  |
| **Age** | -0.03 | 0.02 | 2.22 | 1 | .14 | 0.98 | [0.94, 1.01] |
| **Gender (M=1, F=2)** | -1.35 | 0.55 | 5.99 | 1 | .01 | 0.26 | [0.09, 0.77] |
| **Political ideology** | -0.26 | 0.32 | 0.64 | 1 | .42 | 0.77 | [0.41, 1.45] |
| **Political information sharing** | 0.18 | 0.26 | 0.51 | 1 | .47 | 1.20 | [0.73, 1.99] |
| **Prosocial activism** | -0.26 | 0.25 | 1.11 | 1 | .29 | 0.77 | [0.48, 1.25] |
| **Attack or manipulation of others** | 0.75 | 0.28 | 7.00 | 1 | .01 | 2.11 | [1.21, 3.68] |
| **Entertainment** | 0.33 | 0.21 | 2.38 | 1 | .12 | 1.39 | [0.92, 2.09] |
| **Awareness** | 0.14 | 0.35 | 0.17 | 1 | .68 | 1.16 | [0.58, 2.31] |
| **Political self-expression** | -0.46 | 0.28 | 2.77 | 1 | .10 | 0.63 | [0.37, 1.09] |
| **Fighting false information** | 0.95 | 0.28 | 11.58 | 1 | <.001 | 2.59 | [1.50, 4.47] |
| **Cognitive Perceptual schizotypy** | -0.23 | 0.34 | 0.44 | 1 | .51 | 0.80 | [0.41, 1.55] |
| **Constant** | -1.75 | 2.06 | 0.72 | 1 | .40 | 0.17 |  |

**Table A3.6. Ordinal Regression: Predictors of Amplification of False Stories, Study 2 (all predictors, right wing participants only, *N=*250).**

|  | ***Estimate*** | ***Std. Error*** | ***Wald*** | ***df*** | ***Sig.*** | ***95% C.I. for Estimate*** |
| --- | --- | --- | --- | --- | --- | --- |
| **Age** | -0.05 | 0.02 | 9.35 | 1 | .002 | [-0.07, -0.02] |
| **Gender (M=1, F=2)** | -0.29 | 0.39 | 0.54 | 1 | .46 | [-1.06, 0.48] |
| **Political ideology** | -1.09 | 0.31 | 12.38 | 1 | <.001 | [-1.70, -0.48] |
| **Political information sharing** | 0.08 | 0.21 | 0.16 | 1 | .69 | [-0.32, 0.49] |
| **Prosocial activism** | -0.21 | 0.21 | 0.99 | 1 | .32 | [-0.61, 0.20] |
| **Attack or manipulation of others** | 0.55 | 0.24 | 5.07 | 1 | .02 | [0.07, 1.02] |
| **Entertainment** | 0.05 | 0.17 | 0.08 | 1 | .77 | [-0.28, 0.37] |
| **Awareness** | 0.22 | 0.28 | 0.59 | 1 | .44 | [-0.34, 0.77] |
| **Political self-expression** | -0.04 | 0.23 | 0.04 | 1 | .85 | [-0.49, 0.41] |
| **Fighting false information** | 0.46 | 0.21 | 4.60 | 1 | .03 | [0.04, 0.88] |
| **Cognitive Perceptual schizotypy** | 0.46 | 0.27 | 2.93 | 1 | .09 | [-0.07, 0.99] |
